# Activation loop phosphorylation of a non-RD receptor kinase initiates plant innate immune signaling

**DOI:** 10.1073/pnas.2108242118

**Published:** 2021-09-16

**Authors:** Kyle W. Bender, Daniel Couto, Yasuhiro Kadota, Alberto P. Macho, Jan Sklenar, Paul Derbyshire, Marta Bjornson, Thomas A. DeFalco, Annalise Petriello, Maria Font Farre, Benjamin Schwessinger, Vardis Ntoukakis, Lena Stransfeld, Alexandra M. E. Jones, Frank L. H. Menke, Cyril Zipfel

**Affiliations:** ^a^Institute of Plant and Microbial Biology, Zurich-Basel Plant Science Center, University of Zurich, 8008 Zurich, Switzerland;; ^b^The Sainsbury Laboratory, University of East Anglia, Norwich NR4 7UH, United Kingdom

**Keywords:** receptor kinase, phosphorylation, signaling

## Abstract

Receptor kinases (RKs) are fundamental for extracellular sensing and regulate development and stress responses across kingdoms. In plants, leucine-rich repeat receptor kinases (LRR-RKs) are primarily peptide receptors that regulate responses to myriad internal and external stimuli. Phosphorylation of LRR-RK cytoplasmic domains is among the earliest responses following ligand perception, and reciprocal transphosphorylation between a receptor and its coreceptor is thought to activate the receptor complex. Originally proposed based on characterization of the brassinosteroid receptor, the prevalence of complex activation via reciprocal transphosphorylation across the plant RK family has not been tested. Using the LRR-RK ELONGATION FACTOR TU RECEPTOR (EFR) as a model, we set out to understand the steps critical for activating RK complexes. While the EFR cytoplasmic domain is an active protein kinase in vitro and is phosphorylated in a ligand-dependent manner in vivo, catalytically deficient EFR variants are functional in antibacterial immunity. These results reveal a noncatalytic role for EFR in triggering immune signaling and indicate that reciprocal transphoshorylation is not a ubiquitous requirement for LRR-RK complex activation. Rather, our analysis of EFR along with a detailed survey of the literature suggests a distinction between LRR-RKs with RD- versus non-RD protein kinase domains. Based on newly identified phosphorylation sites that regulate the activation state of the EFR complex in vivo, we propose that LRR-RK complexes containing a non-RD protein kinase may be regulated by phosphorylation-dependent conformational changes of the ligand-binding receptor, which could initiate signaling either allosterically or through driving the dissociation of negative regulators of the complex.

## Significance

Protein kinases lacking Arg in the catalytic loop HxD motif (i.e., non-RD kinases) are associated with innate immune signaling across kingdoms. Phosphorylation activates plant immune receptor kinases (RKs), but the mechanistic details of activation are limited. Using the non-RD immune RK ELONGATION FACTOR TU RECEPTOR (EFR) as a model, we investigated the role of the receptor cytoplasmic domain in immune signaling and found that the catalytic activity of EFR is dispensable for antibacterial immunity. Nevertheless, ligand-induced EFR-mediated signaling is initiated by activation loop phosphorylation, but not via the catalytic activity of the receptor protein kinase domain. We propose that leucine-rich repeat-receptor kinase complexes containing a non-RD kinase are activated through phosphorylation-dependent conformational changes of the receptor cytoplasmic domain.

The translation of extracellular stimuli into intracellular signaling activities is carried out by myriad receptors, localized primarily at the plasma membrane. In metazoans, this role is fulfilled by proteins with diverse molecular architectures, which includes ligand-perceiving G protein-coupled receptors, receptor tyrosine kinases (RTKs), Toll-like receptors, integrins, and ligand-gated ion channels. In plants, plasma membrane-localized receptor kinase (RK) complexes are the primary receivers of extracellular molecular signals, and their importance in environmental adaptation and plant development is underscored by the evolutionary expansion of RK gene families in plant genomes (1–5). RKs are structurally analogous to metazoan RTKs and consist of an extracellular domain that mediates ligand perception and protein–protein interactions, a single-pass transmembrane domain, and a cytoplasmic dual-specificity Ser/Thr and Tyr protein kinase domain (6–8). Of note, plant RK cytoplasmic protein kinase domains share monophyletic ancestry with the well-known Interleukin-1 receptor-associated kinases that have central roles in innate immune signaling in animals (1, 9). Among plant RKs, members with leucine-rich repeat (LRR) ectodomains (LRR-RKs) represent the largest subfamily and fulfill critical roles in development and stress response (3, 10). LRR-RKs have thus been the focus of extensive biochemical and structural analyses aimed at understanding how they activate intracellular signaling in response to ligand perception (11–15). A common mode of activation among RKs is ligand-induced heterodimerization with coreceptors. Following ligand perception, plant LRR-RKs recruit coreceptors—which are themselves LRR-RKs with short, shape-complementary ectodomains—that typically form contacts with both the ligand and the ligand-binding receptor (3). In this context, ligand-dependent receptor/coreceptor heterodimer formation acts as a binary switch to initiate intracellular signaling (11, 13, 14, 16). Although structural analysis of receptor ectodomains has provided a detailed understanding of how receptor/coreceptor interactions occur in a ligand-dependent manner (3, 13–15, 17–19), much less is known mechanistically about how receptor/coreceptor dimerization activates the intracellular protein kinase activities and subsequent downstream signaling.

Early work on the brassinosteroid (BR) receptor BRASSINOSTEROID INSENSITIVE 1 (BRI1), an LRR-RK, established that phosphorylation of both the ligand-binding receptor and coreceptor was critical for activating BR responses (20–23). In vitro analysis of recombinant cytoplasmic domains revealed that BRI1 can phosphorylate its coreceptor, BRI1-ASSOCIATED RECEPTOR KINASE 1 (BAK1, also known as SOMATIC EMBRYOGENESIS RECEPTOR KINASE 3; SERK3), and that BAK1-mediated phosphorylation of BRI1 could enhance BRI1 substrate phosphorylation (22). Based on this, and the observation that both BRI1 and BAK1 are phosphorylated in vivo in a BR-dependent manner, ligand-triggered dimerization was proposed to facilitate reciprocal transphosphorylation between the receptor and coreceptor cytoplasmic domains (22, 24, 25), with phosphorylation events in the activation loop playing a central role. Most eukaryotic protein kinases are Arg-Asp (RD) protein kinases, with Arg in the conserved subdomain VIb catalytic loop HRD motif (26) that require activation loop phosphorylation for catalytic activity (27, 28). Indeed, BRI1 and BAK1 are both RD protein kinases, consistent with the requirement for activation loop phosphorylation for protein function in vivo (20–22, 29, 30). However, several protein kinases, particularly in plants (31, 32), lack the conserved HRD Arg and are known as non-RD protein kinases. In both animals and plants, non-RD protein kinases have been associated with innate immune functions (31, 32). Distinct from the RD-type, non-RD protein kinases are thought not to require activation loop phosphorylation for function (33). Although much less is known mechanistically about how non-RD kinases are regulated, it is clear that their in vitro catalytic activities are low compared to their RD counterparts (34). As such, it is not certain how or whether reciprocal activation loop transphosphorylation would function to activate RK complexes containing at least one non-RD protein kinase.

Targeted analysis of phosphorylation by tandem mass spectrometry (MS/MS) of recombinant or affinity-purified proteins identified a large number of phosphorylation sites throughout LRR-RK cytoplasmic domains (35–47). These targeted studies are complemented by phosphoproteomic analyses, revealing multisite phosphorylation on several RKs in vivo (48–52). By comparison to the number of phosphorylation sites documented in plant and animal systems, the vast majority of sites have not been connected experimentally to biochemical or physiological functions (53). Nevertheless, biochemical and genetic analyses have shed light on the functions of site-specific phosphorylation for some plant RKs. For example, phosphorylation of S891 in the ATP-binding loop of BRI1 inhibits its function, as indicated by increased BR responsiveness in transgenic plants expressing a nonphosphorylatable S891A mutant (54, 55).

Several LRR-RKs are phosphorylated within their intracellular juxtamembrane domains (52), and although the specific functions of these phosphorylation events are unclear, they may control receptor stability and ligand-induced endocytic trafficking (38, 56, 57). In particular, phosphorylation of T705 of the rice LRR-RK XA21 inhibits immune function in vivo (57). This residue is conserved broadly across the *Arabidopsis thaliana* (hereafter, *Arabidopsis*) LRR-RK family, and a variant of FLAGELLIN SENSING 2 (FLS2) carrying a Thr-to-Val mutation at this position (T867V) does not undergo ligand-induced endocytosis (56), suggesting that phosphorylation at this site triggers receptor internalization after initiation of downstream signaling. Additional phosphorylation sites in the XA21 juxtamembrane domain are proposed to control protein stability through inhibition of cleavage by an unknown protease (38). Phosphorylation of S938 in the protein kinase domain of FLS2 positively regulates flg22 responses (39, 46), but it is not clear whether this site is derived from autophosphorylation or is the target of another protein kinase in vivo. Evidence from analysis of the LRR-RK HAESA (HAE), which is involved in floral organ abscission, indicates that RK phosphorylation might also control substrate specificity. The HAE cytoplasmic domain is phosphorylated in vitro on T872 and substitution of Thr-for-Asp (T872D) specifically increases Tyr autophosphorylation activity of the protein (42), highlighting the possibility that site-specific phosphorylation might control the dual-specificity nature of plant RKs. Although it is difficult to draw general conclusions, multiple regulatory phosphorylation sites exist on RKs, suggesting broad cellular capacity to control RK-mediated processes.

BAK1, a common coreceptor for multiple ligand-binding LRR-RKs, is phosphorylated on multiple residues in its catalytic domain and C-terminal tail (22, 30, 36, 43, 44, 47). Interestingly, a cluster of autophosphorylation sites in the BAK1 C-terminal tail (S602, T603, S604, and S612) is important for a subset of BAK1 functions based on the conservation of a specific Tyr residue (which we refer to as the “VIa-Tyr”) in the protein kinase domain of the ligand-binding receptor (47). The BAK1 VIa-Tyr (Y403) is itself phosphorylated in vitro, and mutation to Phe (Y403F) compromises the same subset of BAK1 functions as nonphosphorylatable mutations in the C-terminal tail cluster (47). Although phosphorylation of Y403 or the C-tail cluster is required for full activation of immune responses, the molecular basis for their function is unknown. Intriguingly, several other RKs are phosphorylated on the subdomain VIa-Tyr residue including the LysM-RK CHITIN ELICITOR RECEPTOR KINASE 1 (CERK1) and the B-type lectin S-domain RK LIPOOLIGOSACCHARIDE-SPECIFIC REDUCED ELICITATION (LORE) (58–61). For both CERK1 and LORE, phosphorylation of the VIa-Tyr is required for activation of ligand-induced responses, suggesting a conserved function of this residue in RKs with diverse ectodomain architectures. The LRR-RK ELONGATION FACTOR TU RECEPTOR (EFR) is also phosphorylated on the VIa-Tyr (Y836), and mutation to Phe (Y836F) abolishes ligand-dependent EFR Tyr phosphorylation and downstream signaling, suggesting that Y836 phosphorylation is required for activation of the receptor complex (62). The conservation of VIa-Tyr phosphorylation on plant RKs is intriguing, although no biochemical function has yet been assigned to this important phosphorylation site.

Among the best-described physiological roles for RKs is in activating cell-surface immunity where they function as pattern recognition receptors (PRRs) and perceive pathogen-associated molecular patterns (PAMPs) or host-derived damage-associated molecular patterns (DAMPs) (10, 63). PAMP and DAMP perception sets in motion a battery of signaling events including a BOTRYTIS-INDUCED KINASE 1 (BIK1)-dependent apoplastic oxidative burst, calcium (Ca^2+^) influx (and activation of Ca^2+^-dependent protein kinases), and BIK1-independent initiation of mitogen-activated protein kinase (MAPK) cascades that collectively drive transcriptional reprogramming to ultimately halt pathogen ingress (64–68). In *Arabidopsis*, the LRR-RKs FLS2 and EFR function as PRRs to perceive the PAMPs flagellin (or the derived peptide flg22) and elongation factor thermo-unstable (EF-Tu; or the derived peptide elf18), respectively (69, 70). Both receptors form a ligand-dependent complex with the coreceptor BAK1 or other members of the SERK subfamily (71–74). Phosphorylation of both receptor complex components occurs soon after PAMP perception and is required for downstream signaling (34, 47, 62, 74). EFR and FLS2 are substrates of BAK1, as is the receptor-like cytoplasmic kinase (RLCK) BIK1 (34, 43, 75), suggesting that the majority of early activating phosphorylation events are catalyzed by BAK1, a notion that is further supported by the dominant-negative effect of catalytically inactive BAK1 mutants on PAMP signaling (34, 74).

Because of the exogenous nature of their cognate ligands, PRRs serve as a useful model to understand the biochemical mechanisms regulating receptor activity since it is possible to study acute responses to ligand perception. We previously reported on the unidirectional phosphorylation of EFR by BAK1 in vitro and on the critical role of EFR Tyr phosphorylation in receptor complex activation (34, 62). Building on these previous studies, in the present work we use EFR as a model LRR-RK and a genetic complementation approach to dissect the steps critical for phosphorylation-mediated LRR-RK complex activation. We reveal that EFR protein kinase activity is dispensable for elf18-induced immune signaling and antibacterial immunity and identify phosphorylation sites on purified native EFR that regulate elf18-induced receptor complex activation. Unexpectedly, we discovered EFR activation loop phosphorylation as a critical component of receptor complex activation, indicating that non-RD protein kinases might be regulated in a manner similar to enzymes of the RD type. Collectively, our data challenge the ubiquity of reciprocal transphosphorylation as a requirement for LRR-RK complex activation and support a noncatalytic role for ligand-binding receptors with non-RD intracellular protein kinase domains. We propose a mechanism where phosphorylation-dependent conformational changes of EFR would enhance coreceptor activity—either allosterically or by triggering the dissociation of negative regulators—to initiate signaling downstream of the receptor complex.

## Results

### EFR Phosphorylation in the Receptor Complex Occurs Independently of Its Own Catalytic Activity.

**Fig. 1.**
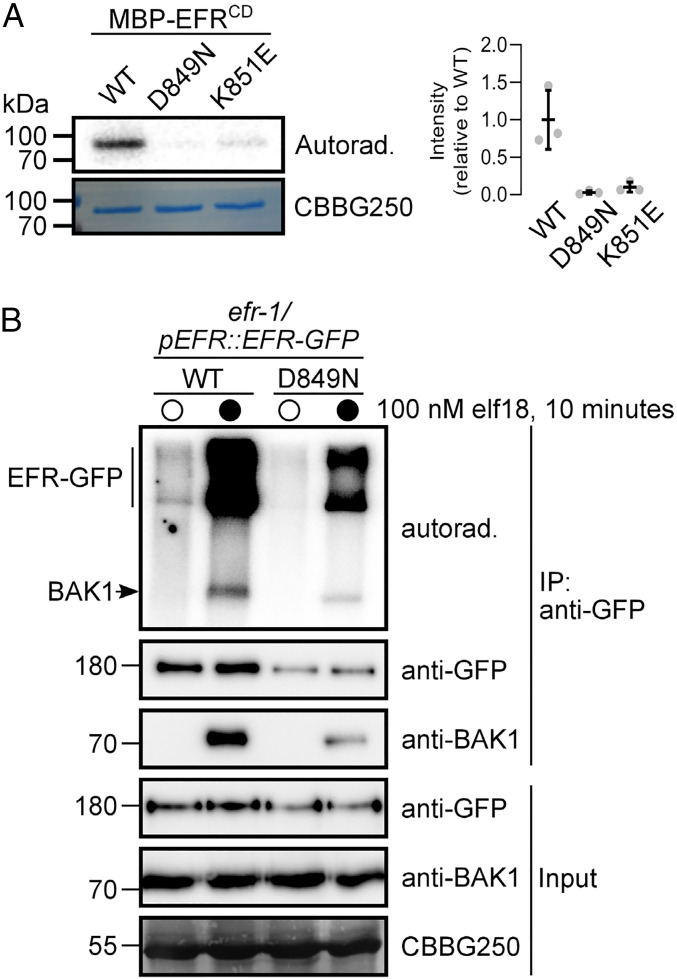
EFR is an active protein kinase but its activity is not required for phosphorylation in an isolated receptor complex. (*A*) In vitro protein kinase activity of recombinant MBP-tagged EFR^CD^ (WT) and catalytic site mutants (D849N and K851E). Recombinant proteins were incubated with 1 µCi γ[^32^P]ATP for 10 min and ^32^P incorporation was assessed by autoradiography. Relative quantification of ^32^P incorporation from three independent assays is shown. (*B*) On-bead kinase activity assay of immunopurified EFR-GFP (mock treatment, open circles) and EFR-GFP/BAK1 (elf18-treated, closed circles) complexes purified with GFP-Trap beads. Bead-bound receptor complexes were incubated with 5 µCi γ[^32^P]ATP for 30 min and ^32^P incorporation was assessed by autoradiography. On-bead kinase activity assays were performed three times with similar results each time. In *A* and *B*, Coomassie stain is shown as loading control (CBBG250).

The cytoplasmic domain of EFR contains a non-RD–type protein kinase domain with Cys (C848) in place of Arg in the catalytic HRD motif, suggesting that the EFR protein kinase domain does not require activation loop phosphorylation for function (33). Nevertheless, the recombinant EFR cytoplasmic domain (EFR^CD^) is capable of autophosphorylation in vitro following purification from *Escherichia coli*, and similar to RD-type protein kinases, mutation of either the proton acceptor to Asn (D849N) or the catalytic loop Lys that participates in substrate coordination (K851E) (76) compromises the protein kinase activity of EFR^CD^ (Fig. 1*A*) (34, 77). We previously observed that an immunopurified EFR-BAK1 complex was catalytically active in vitro (62), and thus we tested whether EFR protein kinase activity was required for in vitro phosphorylation of the native receptor complex. WT EFR or EFR^D849N^ were immunopurified from transgenic *Arabidopsis* seedlings expressing green fluorescent protein (GFP)-tagged EFR variants treated with mock or 100 nM elf18 for 10 min, and the partially purified receptor complexes were then incubated with γ[^32^P]ATP to assess their protein kinase activity. As in previous studies (62), EFR immunopurified from mock-treated seedlings showed minimal phosphorylation relative to the EFR-BAK1 complex purified from elf18-elicited seedlings (Fig. 1*B*). Both BAK1 and EFR were phosphorylated in receptor complexes immunopurified from elf18-treated seedlings. Unexpectedly, the receptor complex containing EFR^D849N^ was still catalytically active, and both EFR^D849N^ and BAK1 were phosphorylated even though lower amounts of protein were immunopurified for EFR^D849N^ versus the WT (Fig. 1*B*). This suggests that EFR catalytic activity is not required for its phosphorylation in the active receptor complex. It is likely that phosphorylation on the EFR^D849N^-containing complex is derived from BAK1 (or related SERKs), but we cannot exclude that other protein kinases in the immunoprecipitate could be responsible.

### EFR Protein Kinase Activity Is Not Required for Immune Signaling.

Because EFR protein kinase activity was not required for its phosphorylation in the isolated active receptor complex, we tested whether different catalytic site mutants of EFR could trigger the elf18-induced oxidative burst following transient expression in *Nicotiana benthamiana*, which lacks a native receptor for this PAMP. Transient expression of EFR confers perception of elf18 in *N. benthamiana* leaves as indicated by an elf18-induced oxidative burst (70) (*SI Appendix*, Fig. S1). Like the WT receptor, both EFR^D849N^ and a second catalytically deficient mutant, EFR^K851E^, could activate an elf18-induced oxidative burst in *N. benthamiana* leaves but with reduced intensity and delayed maxima compared to WT EFR (*SI Appendix*, Fig. S1).

**Fig. 2.**
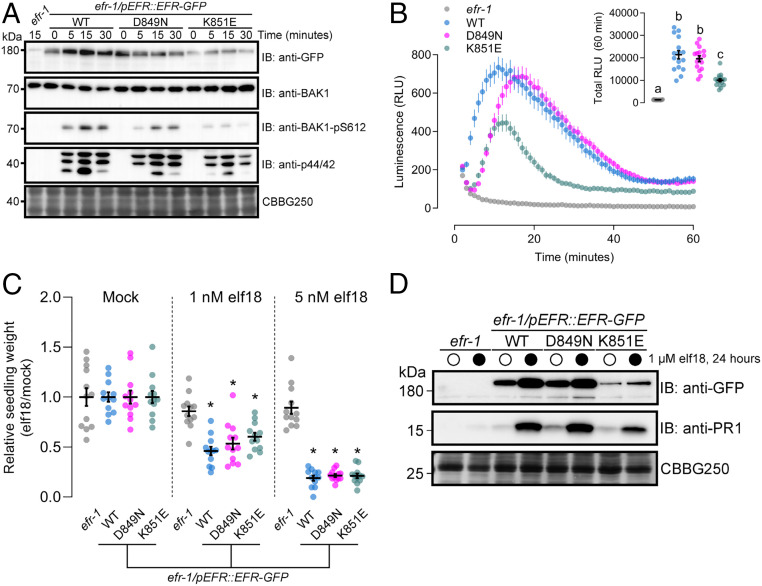
Catalytically inactive EFR variants are competent for elf18-induced PTI signaling. (*A*) Immunoblot analysis of elf18-induced phosphorylation of BAK1 (anti-BAK1-pS612) and MAPKs (anti-p44/42) in 12-d-old seedlings treated with 1 µM elf18 for the indicated time. Anti-GFP shows protein accumulation of EFR and the site-directed mutants. Anti-BAK1 shows similar abundance of the coreceptor across all samples. Coomassie stain is shown as loading control (CBBG250). Blotting experiments were performed three times with similar results. (*B*) Time course of the oxidative burst in leaf discs from transgenic *Arabidopsis* expressing EFR-GFP (WT) or kinase-dead variants (D849N or K851E) in the *efr-1* knockout background induced by treatment with 100 nM elf18. Points are mean with SEM. *Inset* shows mean with SEM of total luminescence over 60 min with individual data points. Means with like letter designations are not statistically different (Kruskal–Wallis ANOVA, *n* = 16 leaf discs, *P* < 0.000001, Dunn’s multiple comparisons test). The experiment was repeated three times with similar results. (*C*) Relative weight of seedlings grown in liquid media for 10 d with (1 or 5 nM) or without (Mock) the addition of elf18 peptide. Mean with SEM and individual values are shown. Asterisk indicates statistical difference from *efr-1* within a given treatment (two-way ANOVA, *n* = 12 seedlings, *P* < 0.0001, Dunnett’s multiple comparison test). The experiment was repeated three times with similar results. (*D*) Accumulation of PR1 protein assessed by immunoblotting with anti-PR1 antibodies 24 h after infiltration of leaves from 3-wk-old plants with mock (open circles) or 1 µM elf18 (closed circles). Coomassie stain is shown as loading control (CBBG250). PR1 accumulation was assessed in three independent experiments with similar results each time.

We next tested whether EFR^D849N^ and EFR^K851E^ could complement the *efr-1* loss-of-function *Arabidopsis* mutant for elf18-induced immune signaling and antibacterial immunity. First, we compared WT and catalytic site mutants of EFR for activation of elf18-induced phosphorylation events by immunoblotting with phosphorylation site-specific antibodies, including phosphorylation of BAK1-S612, which is a marker for receptor complex formation and activation (47), and MAPKs (Fig. 2*A*). In transgenic *Arabidopsis* lines expressing EFR or the corresponding catalytic site mutants, we observed a time-dependent increase of BAK1-S612 and MAPK phosphorylation that peaked at 15 min following stimulation with elf18 (Fig. 2*A*). To understand how BAK1-S612 phosphorylation occurs in the absence of EFR catalytic function, we performed immunoblotting experiments using transgenic seedlings expressing either WT or kinase-dead (D416N) BAK1 in the *bak1-4* background. We observed an elf18-dependent increase in BAK1-S612 phosphorylation in WT but not D416N seedlings (*SI Appendix*, Fig. S2), indicating that S612 is an autophosphorylation site in the active receptor complex, consistent with our observation that EFR catalytic activity is not required for BAK1-S612 phosphorylation. We next measured the oxidative burst in response to elf18 treatment in the same transgenic lines. As was observed in *N. benthamiana*, both catalytic site mutants could activate an elf18-induced oxidative burst similar to the WT receptor, but with reduced intensity or with delayed maxima (Fig. 2*B*). Notably, the total oxidative burst was reduced in transgenic plants expressing EFR^K851E^ compared to either WT or EFR^D849N^ (Fig. 2 *B*, *Inset*); however, this difference might be attributed to reduced accumulation of the receptor in the EFR^K851E^ transgenic line (Fig. 2*A*). Finally, we tested the effect of elf18 on seedling growth over 12 d in our complementation lines. Plants expressing the catalytically inactive variants of EFR were as sensitive to elf18 as the WT line, even at low (1 nM) concentrations of the elicitor (Fig. 2*C*). Collectively, these experiments indicate that catalytic site mutants of EFR are competent to initiate elf18-induced signaling.

As a second measure of long-term plant immunity signaling, we assayed salicylic acid (SA) signaling through accumulation of the SA reporter protein PATHOGENESIS-RELATED GENE 1 (PR1) (78, 79) by immunoblotting with anti-PR1 antibodies. In the WT complementation line, elf18 infiltration into leaves induced robust PR1 accumulation 24 h after treatment (Fig. 2*D*). Like the WT, transgenic plants expressing either EFR^D849N^ or EFR^K851E^ activated PR1 accumulation in response to elf18 treatment. We additionally observed accumulation of EFR in all transgenic lines (Fig. 2*D*), consistent with transcriptional up-regulation of the receptor following elf18 perception (70, 80). The accumulation of PR1 and EFR indicates that elf18-induced transcriptional responses are triggered independently of EFR protein kinase activity.

### EFR Kinase Activity Is Dispensable for Antibacterial Immunity.

**Fig. 3.**
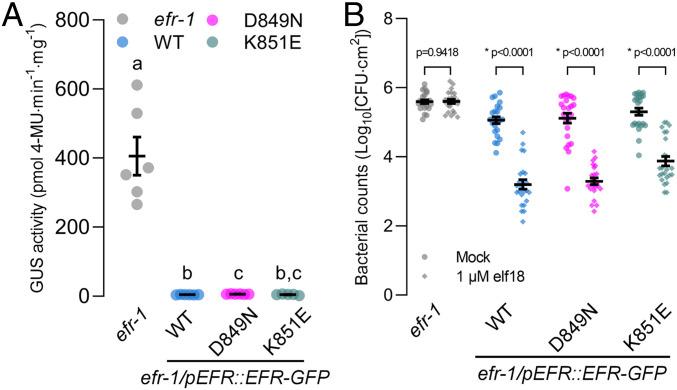
Loss of EFR kinase activity does not compromise immune responses. (*A*) Fluorometric measurement of β-GUS activity in leaves of 3-wk-old plants 5 d after infiltration of leaves with *Agrobacterium* containing the pBIN19g:GUS plasmid. Mean with SEM and individual data points are shown. Means with like letter designations are not statistically different (Brown–Forsythe ANOVA, *P* = 0.000338, *n* = 5 or 6 plants, Dunnett’s multiple comparisons). The experiment was repeated three times with similar results. (*B*) Growth of *Pto* DC3000 2 d after infiltration in leaves pretreated with either mock or 1 µM elf18 for 24 h. Mean with SEM and individual data points (*n* = 23 or 24 plants) from three pooled independent experiments are shown. *P* values are derived from the comparison between elf18 pretreatment and mock, separately for each genotype as described in *Experimental Procedures*. Asterisk indicates a statistical difference between mock and elf18-treated leaves within each genotype.

It is possible that although immune signaling is intact, antibacterial immunity could be compromised without catalytically active EFR in the receptor complex. We therefore tested whether catalytic site mutants of EFR were functional in two different pathogen infection assays: *Agrobacterium*-mediated transient transformation of *Arabidopsis* leaves and elf18-induced resistance to *Pseudomonas syringae* pv. *tomato* DC3000 (*Pto* DC3000) infection (70). In *Arabidopsis*, perception of EF-Tu from *Agrobacterium tumefaciens* suppresses transient transformation (70). To test whether EFR protein kinase activity is required to suppress transient transformation, we infiltrated leaves of *efr-1* or our complementation lines with *Agrobacterium* carrying a binary plasmid containing a β-glucuronidase (GUS) transgene (*Agrobacterium/pBIN19g:GUS*). As a proxy for transformation efficiency, we measured GUS activity in leaf extracts using a quantitative fluorometric assay (81). In the *efr-1* knockout line, we consistently observed GUS activity in extracts from leaves infiltrated with *Agrobacterium/pBIN19g:GUS* (Fig. 3*A*). By comparison, GUS activity in leaf extracts from transgenic plants expressing WT EFR or the catalytic site mutants was roughly 100 times lower. These results indicate that catalytically deficient variants of EFR can restrict *Agrobacterium*-mediated transient transformation of *Arabidopsis* leaves similar to the WT receptor.

Finally, we tested whether elf18 responses triggered by the EFR catalytic site mutants could restrict *Pto* DC3000 infection. To this end, we pressure infiltrated leaves of *efr-1* and the complementation lines with either mock (sterile ddH_2_0) or 1 μM elf18, and then 24 h later pressure-infiltrated *Pto* DC3000. After 2 d, we measured pathogen levels by colony counting (Fig. 3*B*). For *efr-1* knockout mutants, pathogen titer was similar in mock- and elf18-treated plants. In contrast, for the WT and both catalytic site mutant complementation lines, pretreatment of leaves with elf18 resulted in restriction of bacterial replication compared to the mock treatment (Fig. 3*B*), indicating that elf18-induced immune responses triggered by EFR catalytic site mutants were sufficient for induced resistance to *Pto* DC3000.

Collectively, our analysis of short- (reactive oxygen species [ROS], MAPK) and long-term (seedling growth inhibition, PR1 accumulation, transient transformation, induced resistance) immune responses in transgenic plants expressing EFR^D849N^ or EFR^K851E^ demonstrate that elf18-triggered immunity does not require the catalytic activity of its cognate receptor EFR.

### Ser/Thr Phosphorylation Regulates EFR-Mediated elf18 Responses.

Given that EFR is phosphorylated in the active elf18-EFR-BAK1 receptor complex, we aimed to identify the sites of phosphorylation and to test whether phosphorylation regulates elf18 responses in a site-specific manner. To identify phosphorylation sites on EFR, we carried out in vitro protein kinase assays on EFR-GFP immunopurified from transgenic seedlings treated with mock or 100 nM elf18 and subsequently performed phosphorylation site discovery by liquid chromatography-MS/MS. In total, we identified 12 high-confidence Ser and Thr phosphorylation sites distributed throughout the EFR cytoplasmic domain (*SI Appendix*, Table S1) (82). Several of these sites were previously documented as either in vitro EFR autophosphorylation or BAK1 substrate phosphorylation sites (43), and several were documented as in vivo phosphorylation sites in a recent *Arabidopsis* phosphoproteome analysis (49). Interestingly, some of the sites we identified only occurred on the receptor complex immunopurified from elf18-treated seedlings (*SI Appendix*, Table S1), suggesting that they may be involved in the regulation of EFR-mediated immune signaling.

**Fig. 4.**
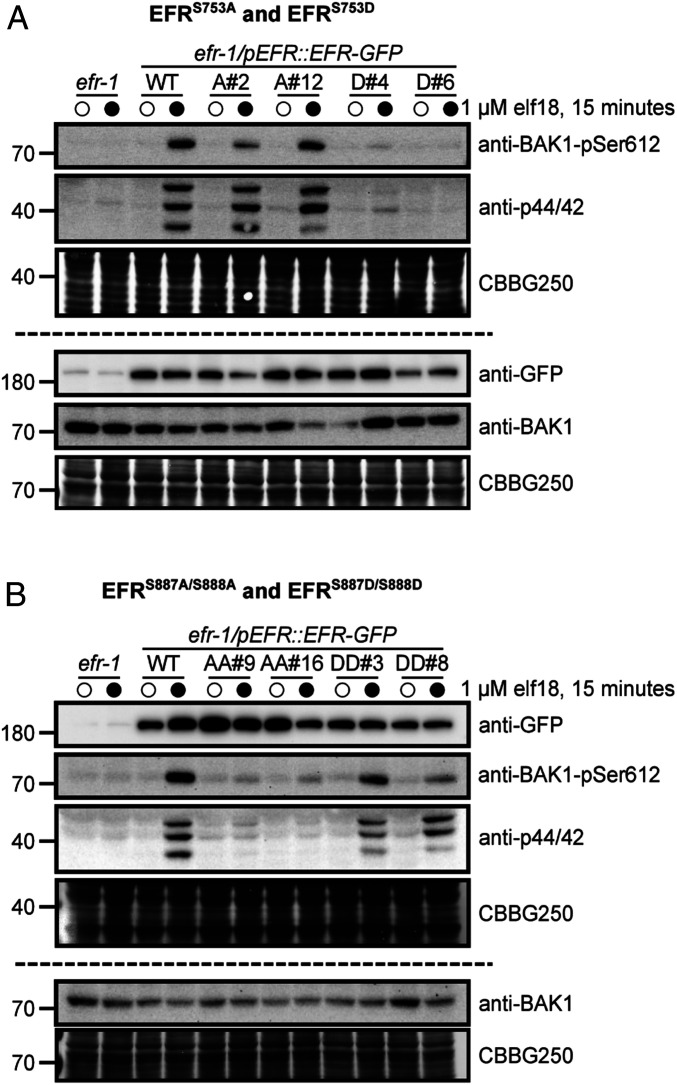
EFR phosphorylation site mutants fail to trigger ligand-induced phosphorylation events. Immunoblot analysis of elf18-induced phosphorylation of BAK1 (anti-BAK1-pS612) and MAP kinases (anti-p44/42) in 12-d-old seedlings expressing WT EFR and (*A*) EFR^S753A^ (A#2, A#12) or EFR^S753D^ (D#4, D#6), or (*B*) EFR^S887A/S888A^ (AA#9, AA#16) or EFR^S887D/S888D^ (DD#3, DD#8) mutants. Seedlings were treated with mock (open circles) or 1 µM elf18 (closed circles) for 15 min. Anti-GFP shows protein accumulation of WT EFR-GFP and the site-directed mutants. Panels above and below the dashed line represent immunoblots derived from replicate SDS/PAGE gels. Coomassie stained blots are shown as loading control (CBBG250). Experiments were repeated three times with similar results.

To test if any of the identified EFR phosphorylation sites regulate elf18-triggered responses, we generated transgenic *Arabidopsis* plants expressing nonphosphorylatable (Ser/Thr-to-Ala) or phospho-mimic (Ser/Thr-to-Asp) mutants of EFR in the *efr-1* background and tested whether the mutants could trigger activation of MAPK cascades in response to elf18 treatment (*SI Appendix*, Fig. S3*A*). Based on this screen, we identified two phosphosite mutants that completely lacked elf18-induced MAPK phosphorylation, namely EFR^S753D^ and EFR^S887A/S888A^. Transgenic plants expressing either the EFR^S887A^ or EFR^S888A^ single-site mutant had reduced but not completely abolished MAPK activation, suggesting that phosphorylation of either residue could fulfill a putative regulatory function. In separate experiments, we tested the capacity of EFR phosphorylation site mutants to trigger BAK1-S612 phosphorylation and confirmed loss of MAPK activation for both the EFR^S753D^ and EFR^S887A/S888A^ receptor variants (Fig. 4). BAK1-S612 phosphorylation could not be detected in crude extracts from transgenics expressing either EFR^S753D^ (Fig. 4*A*) or EFR^S887A/S888A^ (Fig. 4*B*) following elf18 treatment. By comparison, plants expressing EFR^S753A^ and EFR^S887D/S888D^ responded to elf18 similar to the WT complementation lines for both BAK1-S612 and MAPK phosphorylation (Fig. 4). Importantly, neither the transgenic expression of EFR^S753A^ or EFR^S887D/S888D^ led to constitutive MAPK phosphorylation, indicating that both mutant receptors still require ligand-triggered dimerization with BAK1 to activate downstream signaling.

**Fig. 5.**
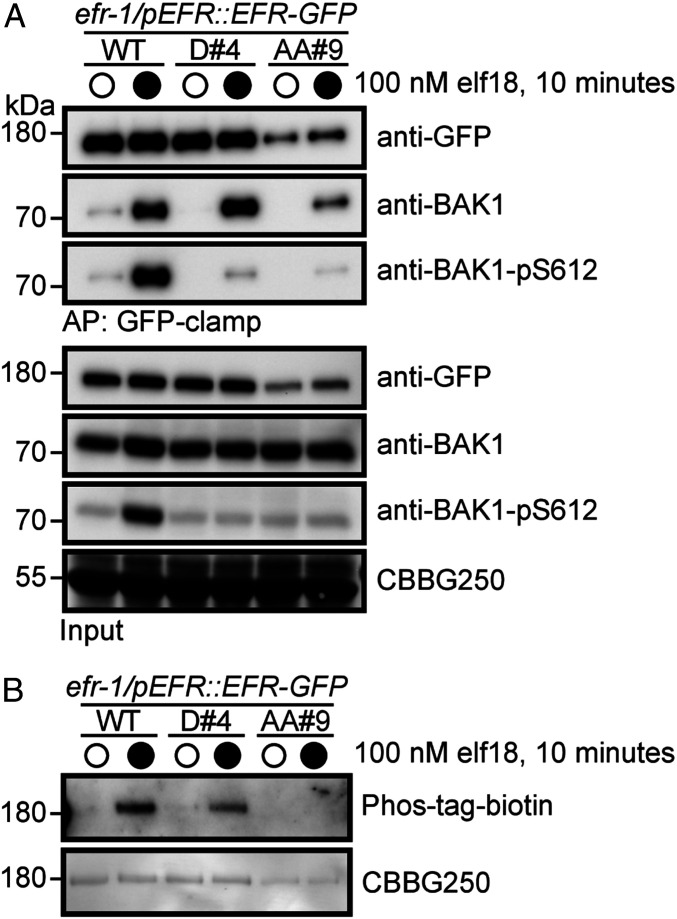
EFR phosphorylation site mutants form a ligand-induced complex with BAK1. (*A*) Immunoblot analysis of elf18-induced receptor complex formation in 12-d-old seedlings expressing either WT EFR or phosphorylation site mutants (S753D, D#4; S887A/S888A, AA#9). Seedlings were treated with either mock (open circles) or 100 nM elf18 (closed circles) for 10 min, followed by coimmunoprecipitation with GFP-clamp beads and blotting with antibodies as indicated. (*B*) Analysis of in vivo phosphorylation of WT EFR or phosphorylation site mutants. Seedlings were treated with either mock (open circles) or 100 nM elf18 (closed circles) for 10 min, followed by immunoprecipitation of GFP-tagged receptors with GFP-Trap beads. Phospho-proteins were detected using a Zn^2+^-Phos-tag::biotin-Streptavidin::HRP complex. In both panels, Coomassie stain is shown as a loading control (CBBG250). Experiments in *A* and *B* were repeated four times with similar results.

Next, we tested whether the EFR^S753D^ and EFR^S887A/S888A^ mutants could form functional ligand-induced receptor complexes (Fig. 5). Coimmunoprecipitation experiments indicated that both EFR^S753D^ and EFR^S887A/S888A^ can form a ligand-induced complex with the coreceptor BAK1 (Fig. 5*A*). However, by comparison to WT EFR, BAK1 copurified with either EFR^S753D^ or EFR^S887A/S888A^ had reduced levels of S612 phosphorylation (Fig. 5*A*), indicating that phosphorylation of S753 and S887/S888 regulate activation of the PRR complex. Additionally, we evaluated the global phosphorylation status of immunopurified EFR or the phosphorylation site mutants by blotting with the biotinylated PhosTag reagent. We could detect elf18-inducible phosphorylation of WT EFR and the EFR^S753D^ mutant, but not the EFR^S887A/S888A^ mutant (Fig. 5*B*), suggesting a strict requirement of EFR activation loop phosphorylation for complex activation.

**Fig. 6.**
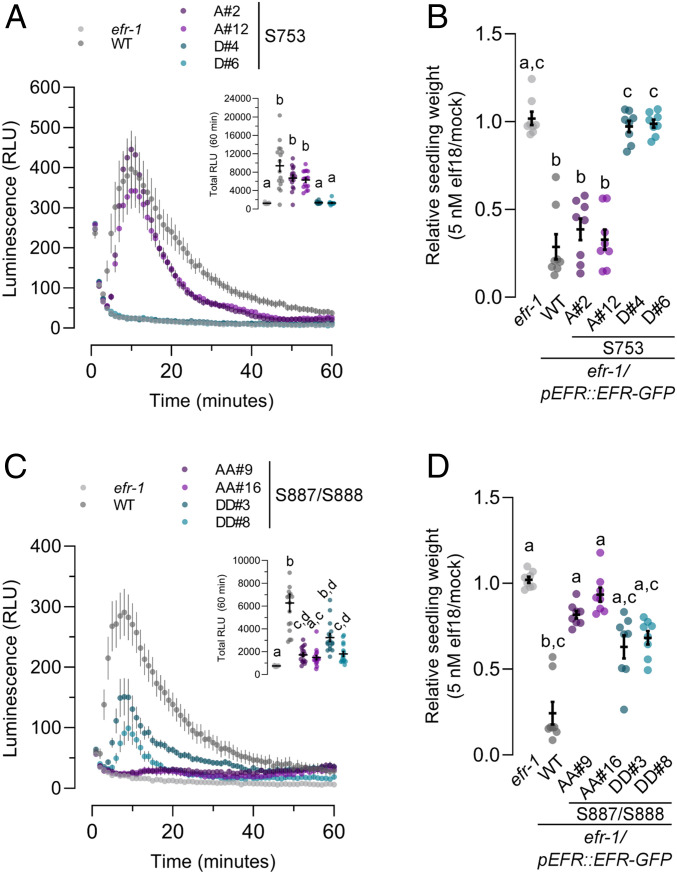
Analysis of PTI responses in EFR phosphorylation site mutants. (*A* and *C*) Oxidative burst in leaf discs from the indicated genotype after treatment with 100 nM elf18. Points represent mean with SEM. *Inset* shows mean with SEM of total luminescence over 60 min. Means with like letter designations are not statistically different (Kruskal–Wallis ANOVA, *n* = 16 leaf discs, *P* < 0.000001, Dunn’s multiple comparisons test). (*B* and *D*) Seedling growth of the indicated genotypes in the presence of 5 nM elf18. Data are shown relative to mock treated seedlings for each genotype. Individual data points with mean and SEM are shown. Means with like letter designations are not statistically different (*B*, Kruskal–Wallis ANOVA, *P* = 0.00001, *n* = 8 seedlings, Dunn’s multiple comparison test; *D*, Kruskal–Wallis ANOVA, *P* < 0.000001, *n* = 8 seedlings, Dunn’s multiple comparison test). All experiments presented were repeated three times with similar results.

Finally, we hypothesized that specific EFR phosphorylation sites might regulate distinct downstream pathways in a manner reminiscent of animal RTKs (83). We therefore tested whether the EFR^S753D^ and EFR^S887A/S888A^ mutants were compromised in other branches of immune signaling or whether MAPK activation was the only downstream response affected. Based on our observations of receptor complex phosphorylation, we expected that other downstream responses would be similarly abolished in transgenic plants expressing either EFR^S753D^ or EFR^S887A/S888A^. Indeed, for the apoplastic oxidative burst (Fig. 6 *A* and *C*) and seedling growth inhibition (Fig. 6 *B* and *D*), both phosphorylation site mutants were blind to elf18 treatment, suggesting that phosphorylation of S753 or S887/S888 does not function to regulate specific branches of immune signaling. Unlike MAPK phosphorylation, the S887D/S888D receptor variant did not fully complement *efr-1* mutants for the apoplastic oxidative burst or for seedling growth inhibition (Fig. 6 *C* and *D*), suggesting that the double Asp mutant does not completely mimic for activation loop phosphorylation. In contrast to EFR^S753D^, transgenic plants expressing EFR^S753A^ were not differentially sensitive compared to the WT at low concentrations of elf18 in seedling growth inhibition assays (*SI Appendix*, Fig. S4). In these experiments, we occasionally observed reduced sensitivity of plants expressing EFR^S753A^ to 0.2 nM elf18; however, this effect was small, and was neither consistent between independent transgenic lines nor across independent experiments. Collectively, the loss of elf18 responses in EFR^S753D^ and EFR^S887A/S888A^ mutants indicates that the novel S753 and S887/S888 phosphorylation sites of EFR are negative and positive regulators of receptor complex activation, respectively.

## Discussion

The activation of transmembrane receptors in response to exogenous and endogenous signals is a critical biochemical process in all aspects of organismal development and stress response. The plant plasma membrane is decorated with a diverse suite of RKs that perceive a wide range of ligands. The largest family of RKs in plants, the LRR-RKs, fulfill critical roles in plant development and environmental response. Members of the LRR-RK family function coordinately with coreceptors from the SERK family to activate intracellular signaling following ligand perception. While the details of ligand perception have been quantitatively described (3, 12–15, 18), much less is known about how a switch from the ligand-free state to a ligand-bound activated state triggers intracellular signal transduction via the cytoplasmic protein kinase domains of the receptor and coreceptor.

In the present study, we aimed to understand the requirements for activation of LRR-RK–mediated signaling on the cytoplasmic side of the receptor complex. Using EFR as a model LRR-RK, our analyses reveal that contrary to previous reports (77, 84), catalytic activity of the ligand binding receptor is dispensable for downstream signaling. Although the consensus view is that ligand-induced dimerization triggers reciprocal transphosphorylation of receptor cytoplasmic domains, several lines of evidence suggest that transphosphorylation between the receptor and coreceptor is not required for signaling downstream of elf18 perception. First, the recombinant BAK1 cytoplasmic domain can phosphorylate the EFR cytoplasmic domain in vitro, but not vice versa (34, 43). Second, expression of a BAK1 kinase-inactive mutant in the null *bak1-4* background has a dominant negative effect on the elf18-induced oxidative burst (34), indicating an absolute requirement for the kinase activity of BAK1 (and most likely related SERKs) for the elf18 response and suggesting that the activity of EFR is not sufficient for elf18-triggered signaling. Third, BAK1 phosphorylates the BIK1 activation loop on T237 that is required for BIK1 function (46, 75), and BIK1 is the direct executor for multiple branches of immune signaling (66–68, 77, 85). It is also noteworthy that FLS2 does not phosphorylate BIK1 in vitro (46), and although it has been proposed that EFR-mediated phosphorylation of BIK1 is important for immunity (77), our analysis of EFR kinase-inactive mutants indicates that this is not required for a fully functional immune response *in planta*. Collectively, these prior observations suggest that a simple phosphorylation cascade initiated by BAK1 would be sufficient to activate immunity, and that reciprocal transphosphorylation by both receptor components is not required.

Our observation that the catalytic activity of EFR is dispensable for all elf18-induced immune responses (Figs. 2 and 3) argues against the ubiquity of reciprocal transphosphorylation as an activating mechanism within the plant RK family, even though formation of receptor complexes with multiple protein kinase domains is common (10). One possibility is that different activation mechanisms operate in RK complexes where both partners are RD protein kinases versus those where one partner is a non-RD protein kinase, such as the case for EFR. Although the functional significance is unknown, it is interesting that non-RD identity is broadly conserved in subfamily XII LRR-RKs that are hypothesized to function as PRRs (4, 31). Among reports that we could find in the published literature, with only a few notable exceptions plant RKs with RD-type intracellular protein kinase domains require their catalytic activity for function (*SI Appendix*, Table S2). By comparison, a catalytic mutant of XA21—a non-RD PRR from rice—confers partial immunity to *Xanthomonas oryzae* pv. oryzae (86). FLS2 is reported to require its protein kinase activity for function (87–90); however, this conclusion is ambiguous since the accumulation of kinase-dead FLS2 at the protein level was not evaluated in most cases. Indeed, we previously reported that EFR expressed in *N. benthamiana* under the 35S promoter requires its kinase activity to support elf18-induced ROS, but information on expression of the catalytic mutant was lacking (34). In the present work, we observe clear accumulation of both EFR^D849N^ and EFR^K851E^ associated with complementation of the *efr-1* mutant. The apparent requirement of FLS2 and EFR catalytic activity for pattern-triggered immunity (PTI) signaling reported in previous studies may thus be consequence of poor receptor accumulation under transient expression or in stable transgenic lines. Collectively, this suggests that the dispensibility of catalytic function might be a common feature of non-RD protein kinases that function in immunity.

In the absence of a direct catalytic role, we foresee two possible functions for EFR in the receptor complex. First, EFR could serve as a protein–protein interaction scaffold to define specificity in activating downstream responses. In support of this, studies of chimeric receptor kinases indicate that the cytoplasmic domain of the ligand binding receptor defines signaling specificity (11, 16). This suggests that the EFR cytoplasmic domain functions as a scaffold for the components required to execute immunity-specific downstream signaling. Second, besides functioning as a scaffold, the EFR cytoplasmic domain might serve to allosterically regulate BAK1 catalytic activity in the ligand-bound receptor complex. In either case, EFR phosphorylation could serve as a critical switch to activate the receptor complex and subsequent downstream events.

Even though EFR kinase activity is not required, EFR phosphorylation is critical for immune signaling (62). Here, we identified three regulatory phosphorylation sites on EFR, namely S753 and the S887/S888 doublet. In transgenic plants expressing either EFR^S753D^ or EFR^S887A/S888A^ we observed a loss of both BIK1-dependent (oxidative burst) and BIK1-independent (MAPK) signaling events, suggesting that the defect for both mutants occurs at the level of receptor complex activation. Interestingly, both the S753 and S887/S888 phosphorylation sites localize to subdomains that are important for regulatory conformational dynamics of protein kinases (27, 91, 92). Specifically, S753 is positioned within the αC-helix and the S887/S888 doublet within the activation loop (*SI Appendix*, Fig. S3 *B* and *C*). These residues are well conserved in *Arabidopsis* subfamily XIIa LRR-RKs and in PLANT ELICITOR PEPTIDE 1 RECEPTOR 1 (PEPR1), all of which are known or hypothetical PRRs (4, 31, 32), but not in closely related subfamily XIIb members or other RD-type LRR-RKs (*SI Appendix*, Fig. S3*B*), suggesting that these sites might be important in regulating immune signaling. Activation loop phosphorylation serves as a key regulatory switch of RD protein kinases (27, 93–95), and although a few non-RD protein kinases from nonplant eukaryotes are phosphorylated on their activation loops (96–98), the functional significance of these phosphorylation events is not always well understood.

In a typical RD protein kinase, activation loop phosphorylation triggers conformational changes that establish a catalytically competent active state of the protein kinase domain (99). Based on our observation that catalytic activity is not required for EFR function, we do not think that phosphorylation of S887/S888 is required to promote EFR-mediated catalysis per se, but that an active-like conformation associated with activation loop phosphorylation might function in feed-forward allosteric activation, or might trigger dissociation of negative regulators of the complex (100). Consistent with a possible allosteric mechanism, a complex containing EFR^S887A/S888A^ is largely devoid of any phosphorylation (Fig. 5), including on BAK1-S612. This suggests that phosphorylation of the EFR activation loop precedes all or most other phosphorylation on the receptor complex and that phosphorylation of EFR is required to fully activate BAK1.

Like the EFR^S887A/S888A^ nonphosphorylatable mutant, an EFR^S753D^ phospho-mimic mutant also abolished elf18 responses, but not complex formation with BAK1 (Figs. 4–6). However, distinct from the EFR^S887A/S888A^, elf18-induced phosphorylation of EFR^S753D^ was similar to the WT (Fig. 5), indicating residual protein kinase activity in the complex and phosphorylation of other sites on EFR. Interestingly, S753 is located at the N-terminal end of the ɑC-helix in the protein kinase N-lobe, a region of the protein kinase domain associated with conformational changes that mediate protein kinase activation (92). Consistent with the requirement for EFR-BAK1 complex formation, EFR^S753A^ mutants did not display constitutive activation of any PTI responses. Although a possible mechanism to explain the impact is less clear compared to S887/S888, S753 phosphorylation could disrupt order–disorder transitions of the EFR ɑC-helix, explaining impaired activation of the EFR^S753D^-containing receptor complex. Indeed, intrinsic ɑC-helix disorder can promote an inactive state of some protein kinases, including plant RKs (101, 102), lending support to this notion.

**Fig. 7.**
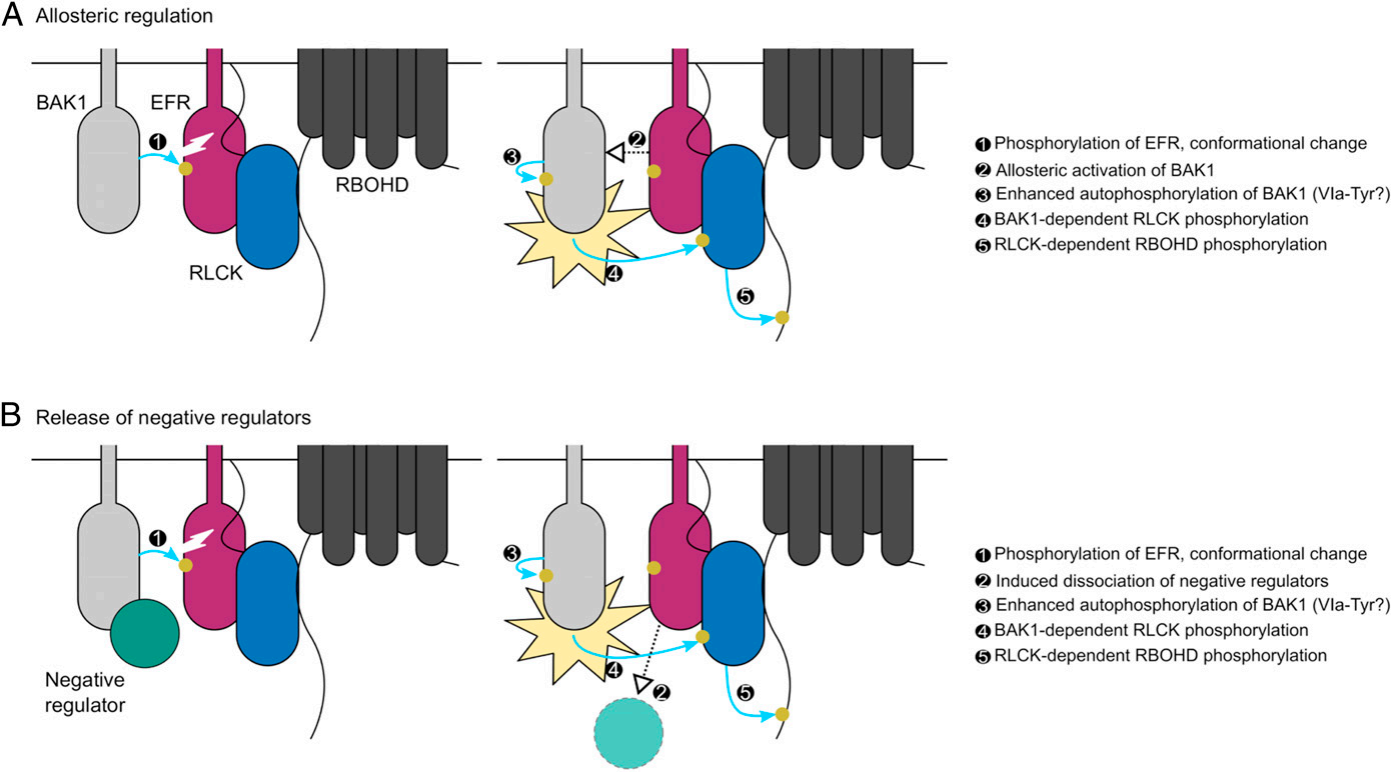
Potential mechanisms for phosphorylation-mediated activation of plant non-RD LRR-RK complexes. Ligand-triggered dimerization promotes phosphorylation of the EFR (purple) activation loop by BAK1 (light gray), inducing a conformational change of the EFR cytoplasmic domain. This conformational rearrangement feeds forward on BAK1 to enhance its catalytic activity either: (*A*) by direct allosteric activation of BAK1 or (*B*) by triggering the release of negative regulators (teal) of BAK1 activation. Either scenario permits full phosphorylation of the complex including on the VIa-Tyr residues. After full activation, BAK1 can phosphorylate the executor RLCKs (blue) to initiate downstream signaling, for example the RBOHD (dark gray)-dependent apoplastic oxidative burst. Yellow circles and blue arrows represent simplified requirements for activation of RBOHD-dependent ROS production by phosphorylation.

Collectively, identification and characterization of EFR phosphorylation sites in the present work and in previous work from our laboratory suggests that phosphorylation-dependent conformational changes of the EFR cytoplasmic domain regulate receptor complex activation. We propose a model (Fig. 7) where initial activation of the complex would occur as a consequence of EFR activation loop phosphorylation triggered by ligand-induced dimerization of EFR and BAK1. Subsequent conformational rearrangement of EFR would enhance BAK1 catalytic activity and promote VIa-Tyr phosphorylation of both complex components either allosterically, or by promoting the dissociation of components that negatively regulate BAK1. Direct phosphorylation of the ɑC-helix would fulfill an inhibitory role, and it is likely that the kinetics of S753 phosphorylation are important for this function. Importantly, both models explain the lack of requirement for the catalytic activity of the ligand-binding receptor. Alternative models for activation of LRR-RK complexes containing a non-RD protein kinase await further testing through a combination of time-resolved quantitative (phospho-)proteomics, homology-guided mutagenesis, and structural biology.

## Experimental Procedures

Detailed experimental procedures used in this study including plant growth conditions, PAMP treatments, cloning and plant transformation, recombinant protein expression and purification, protein extraction, in vitro protein kinase assays, SDS/PAGE and immunoblotting, pathogen infection assays, coimmunopurification, and homology modeling are available as *SI Appendix, Experimental Procedures*. Oligonucleotide sequences used for cloning are available in *SI Appendix*, Table S3 and details of antibodies and immunoblotting conditions are available in *SI Appendix*, Table S4. All materials and detailed protocols are available on request from the corresponding author.

## Supplementary Material

Supplementary File

## Data Availability

The mass spectrometry proteomics data have been deposited to the ProteomeXchange Consortium via the PRIDE (103) partner repository with the dataset identifier PXD025597.

## Change History

April 27, 2022: The title has been updated to correct a typo.
