# Supplementary material for: Activation loop phosphorylation of a non-RD receptor kinase initiates plant innate immune signaling
Source: Proc Natl Acad Sci U S A. 2021 Sep 16;118(38):e2108242118. doi: 10.1073/pnas.2108242118 (PMC8463890; doi:10.1073/pnas.2108242118)
Supplement: Supplementary File [file pnas.2108242118.sapp.pdf]

SI PDF Appendix for

Activation loop phosphorylation of a non-RD receptor kinase initiates plant innate immune signaling

Kyle W. Bender[a,b], Daniel Couto[b,1], Yasuhiro Kadota[b,2], Alberto P. Macho[b,3], Jan Sklenar[b], Paul Derbyshire[b], Marta Bjornson[a,b], Thomas A. DeFalco[a,b], Annalise Petriello[b,4], Maria Font Farre[b,5], Benjamin Schwessinger[b,6], Vardis Ntoukakis[b,7], Lena Stransfeld[a,b], Alexandra M.E. Jones[b,7], Frank L.H. Menke[b], Cyril Zipfel[a,b,8]

a Institute of Plant and Microbial Biology, Zurich-Basel Plant Science Center, University of Zurich, 8008 Zurich, Switzerland.
b The Sainsbury Laboratory, University of East Anglia, Norwich Research Park, NR4 7UH, Norwich, United Kingdom.

1 Current address: Creoptix AG, 8820 Wädenswil, Switzerland.
2 Current address: RIKEN Center for Sustainable Resource Science (CSRS), Plant Immunity Research Group, Yokohama, 230-0045, Japan.
3 Current address: Shanghai Center for Plant Stress Biology, CAS Center for Excellence in Molecular Plant Sciences, Chinese Academy of Sciences, 201602, Shanghai, China.
4 Current address: iQ Biosciences, Berkeley, California, United States, 94710
5 Current address: Department of Plant Sciences, South Parks Road, University of Oxford, Oxford OX1 3RB, United Kingdom
6 Current address: Research School of Biology, The Australian National University, Acton ACT 2601, Australia
7 Current address: School of Life Sciences, Gibbet Hill Road, University of Warwick, Coventry CV4 7AL, United Kingdom

8 To whom correspondence should be directed: cyril.zipfel@botinst.uzh.ch

Experimental Procedures

Plant material, growth conditions, and PAMP treatment

All genetic materials used in this study are in the Col-0 background. Complementation experiments were carried out in the *efr-1* T-DNA insertional mutant (1). For PAMP-induced phosphorylation (BAK1-pS612, MAPK), IP kinase, and seedling growth inhibition assays, seeds were germinated on plates containing 0.5x Murashige and Skoog (MS) basal salt mixture with 1 % (w/v) sucrose and 0.9 % (w/v) phytoagar. Growth conditions for sterile plant culture were: 120 $\mu mol \cdot s^{-1} \cdot m^{-2}$ illumination, 16 hour/8 hour day/night cycle, and a constant temperature of 22 °C. After four days of growth on agar plates, seedlings were transferred to 6- (IP kinase), 24- (PAMP-induced phosphorylation), or 48-well (seedling growth inhibition) sterile culture plates containing liquid 0.5x MS with 1 % (w/v) sucrose. For seedling growth inhibition, liquid media was supplemented with either mock (sterile ultrapure water) or elf18 peptide at the concentrations indicated in figure legends. For all experiments, seedlings were grown in liquid culture for 12 days. For PAMP-induced phosphorylation, the growth media was removed by inverting the plate on a stack of clean paper towel. Seedlings were then treated with fresh MS containing 1 µM elf18 by addition of the PAMP solution directly to the plate for the times indicated in the figures. Treated seedlings (two per treatment/time point) were dried with clean paper towel, transferred to 1.5-mL tubes, and snap-frozen in liquid nitrogen. For IP-kinase assays, seedlings from two 6-well plates (roughly 3.5 g of tissue) were transferred to 50-mL beakers containing MS and were allowed to rest for 1 hour prior to PAMP treatment. The media was then decanted and fresh MS containing mock or 100 nM elf18 was added to the beaker and was infiltrated into seedlings by the application of vacuum for 2 minutes. Seedlings were incubated in the PAMP solution for an additional 8 minutes (10 minutes

treatment total) before drying with clean paper towel and snap-freezing in liquid nitrogen. All PAMP-treated plant materials were stored at -80 °C until use.

For experiments using adult (3- to 4-week-old) plants (oxidative burst, PR1 accumulation, induced resistance, transient transformation), seeds were germinated on soil and plants were grown at 22 °C/20 °C day/night temperatures with 150 $\mu mol \cdot s^{-1} \cdot m^{-2}$ illumination under a 10 hour/14 hour day/night cycle. Plants were watered automatically for 10 minutes three times per week.

Critical reagents

Synthetic elf18 peptide was produced by SciLight Biotechnology (Beijing, China). Peptides were dissolved in sterile ddH_20 to a concentration of 10 mM and stored at -20 °C. Working concentrations were freshly prepared as dilutions from the stock immediately before use.

Cloning and plant transformation

For recombinant protein expression, the EFR cytoplasmic domain was PCR subcloned from Arabidopsis cDNA using primers (Supplementary Table S3) to add KpnI and BamHI restriction sites at the 5' and 3' end of the amplicon, respectively. PCR products and pMAL-c4E plasmid were digested with KpnI and BamHI, digested backbone was treated with calf intestine alkaline phosphatase (CIP), and then digested PCR product and CIP-treated vector backbone were ligated with T4 DNA ligase (New England Biolabs). Ligation reactions were transformed into chemically competent *E. coli* DH10b. Individual colonies were selected for further culturing and plasmid isolation. All constructs were confirmed by DNA sequencing.

For complementation of the *efr-1* mutant with catalytically inactive and phosphorylation site variants of EFR, the EFR promoter (2.4 kb upstream of the start codon) was amplified from genomic DNA and the coding sequence from cDNA using primers for InFusion cloning (Supplementary Table S3). All constructs were confirmed by DNA sequencing prior to transformation into *Agrobacterium tumefaciens* strain GV3101. Plant transformation was carried out using the floral dip method (2). Transformants were selected on MS-agar plates containing 10 µg/mL phosphinothricin.

Site-directed mutagenesis to generate the catalytic site and phosphorylation site mutants was performed by rolling-circle mutagenesis using Phusion polymerase (New England Biolabs) with primers indicated in Supplementary Table S3. All mutagenized constructs were analyzed by DNA sequencing to confirm the presence of the desired mutation and the absence of off-target mutations.

Recombinant protein expression and purification

pMAL-c4E vectors carrying in-frame fusions of the EFR cytoplasmic domain with the N-terminal maltose-binding protein (MBP) tag were transformed into Rosetta 2 cells (NEB) for recombinant protein expression. A single colony was used to inoculate a 15-mL lysogeny broth (LB) starter culture containing 100 µg/mL carbenicillin and was grown overnight at 37 °C with shaking. The next day, 1 L of LB containing 20 mM glucose and 100 µg/ml carbenicillin was inoculated with 10 mL of starter culture and was grown at 37 °C with shaking to an OD_{600} of 0.6. Recombinant protein expression was induced by the addition of 0.3 mM isopropyl β-D-1-thiogalactopyranoside (IPTG) overnight at 18 °C. Cells were pelleted by centrifugation at 5,000 rpm for 15 minutes and were then suspended in

buffer containing 50 mM HEPES-NaOH pH 7.2, 100 mM NaCl, 5 %(v/v) glycerol and cOmplete EDTA-free protease inhibitor tablets (Roche).

Cells were lysed by freeze-thaw followed by sonication (four 20 second cycles with 40 second rests) and lysates were clarified by centrifugation at 35,000 x *g* for 30 minutes at 4 °C. Supernatants were adjusted to 300 mM NaCl and 2 mM DTT and were incubated with 500 µL of amylose resin (New England Biolabs) pre-equilibrated with binding buffer (50 mM HEPES-NaOH pH 7.2, 300 mM NaCl, 5 %(v/v) glycerol, 2 mM DTT) for 1 hour at 4 °C with gentle mixing. The resin was centrifuged for 10 minutes at 500 x *g* and the supernatant was discarded. The resin was suspended in 10 ml of binding buffer, mixed briefly, and centrifuged for 2 minutes at 500 x *g*. This process was repeated for a total of three washes. Bound protein was eluted from the resin by incubation for 15 minutes at 4 °C with mixing in binding buffer containing 20 mM maltose. As a final purification step, proteins eluted from amylose resin were applied to a Superdex 75 Increase size exclusion column pre-equilibrated with 50 mM HEPES-NaOH pH 7.2, 100 mM NaCl, 5 %(v/v) glycerol. Protein purity was assessed by SDS-PAGE and the concentration of peak fractions was determined by the Bradford method using bovine serum albumin as standard. Proteins samples were aliquoted and stored at -80°C until use.

Protein extraction from plant tissues

For analysis of PAMP-induced phosphorylation by immunoblotting (MAPK and BAK1-S612), seedlings frozen in 1.5-mL tubes were pulverized with a nitrogen-cooled plastic micropestle. One hundred microliters per seedling (200 µL total) of extraction buffer containing 50 mM Tris-HCl pH 7.5, 150 mM NaCl, 2 mM EDTA, 10 %(v/v) glycerol, 2 mM DTT, 1 %(v/v) Igepal, and protease and phosphatase inhibitors (equivalent to Sigma-

Aldrich plant protease inhibitor cocktail and phosphatase inhibitor cocktails #2 and #3) was added to each tube and the tissue was ground at 2000 rpm using an overhead mixer fitted with a plastic micropestle. The tubes were centrifuged at 15,000 x *g* for 20 minutes at 4 °C in a refrigerated microcentrifuge. After centrifugation, 150 µL of extract was transferred to a fresh 1.5-mL tube. Protein sample concentrations were normalized using a Bradford assay. Samples were prepared for SDS-PAGE by heating at 80 °C for 10 minutes in the presence of 1X Laemmli loading buffer and 100 mM DTT.

For co-immunoprecipitation and IP kinase assays, approximately 3.5 g of frozen tissue was ground to a fine powder under liquid nitrogen in a nitrogen-cooled mortar and pestle and then further ground with sand in extraction buffer (as described above) at a ratio of 4 mL of buffer per gram of tissue. Extracts were filtered through two layers of Miracloth and centrifuged at 25,000 x g for 30 minutes at 4 °C to generate a clarified extract.

In vitro *protein kinase assays*

To assess the activity of recombinant MBP-EFRCD, 500 ng of purified protein was incubated in a 20-µL reaction with 1 µCi of γ^{32}P-ATP, 2.5 mM each $MgCl_2$ and $MnCl_2$, and 10 µM ATP in 50 mM HEPES-NaOH pH 7.2, 100 mM NaCl, and 5 %(v/v) glycerol for 10 minutes at 30 °C. Reactions were stopped by the addition of Laemmli SDS-PAGE loading buffer and heating at 80 °C for 5 minutes. Reactions were separated by SDS-PAGE followed by transfer to PVDF, and exposure of storage phosphor-screen for 30 minutes. Exposed screens were imaged using an Amersham Typhoon (GE Lifesciences). Image analysis for relative quantification of ^{32}P incorporation was carried out using the ImageQuant software package, with local averaging for background subtraction.

SDS-PAGE, Immunoblotting, and chemiluminescence imaging

Proteins were separated in either 10 %(v/v) (MAPK phosphorylation), 8 %(v/v) (CoIP), or 15 %(v/v) (PR1 accumulation) polyacrylamide gels at 120 V for 95 minutes. Proteins were transferred to PVDF membranes at 100 V for 90 minutes at 4 °C followed by blocking for 2 hours at room temperature or overnight at 4 °C in 5 %(w/v) milk in Tris-buffered saline (50 mM Tris-HCl pH 7.4, 150 mM NaCl; TBS) containing 0.1 %(v/v) Tween-20 (TBS-T). Blots were probed in primary antibody according to the conditions in Supplementary Table S4, followed by washing 4 times for 10 minutes each in TBS-T. When required, blots were then probed in a 1:10,000 dilution of goat-anti-rabbit-HRP conjugate for 30 minutes to 1 hour, followed by washing 3 times for 5 minutes each in TBS-T. Blots were then washed for 5 minutes in TBS and treated with either standard ECL substrate or SuperSignal West Femto high sensitivity substrate (ThermoFisher Scientific). Blots were imaged using a Bio-Rad ChemiDoc Imaging System (Bio-Rad Laboratories). All raw images were saved in the Bio-Rad .scn format and blots were exported as 600 dpi TIFFs for preparation of figures.

For the experiments presented in Figure 2A and Figure 5A, immunoblots probed with anti-BAK1 pS612 antibodies were stripped by incubation in stripping buffer containing 214 mM glycine pH 2.2, 0.1 %(w/v) SDS, 1 %(v/v) Tween-20 4 times for 10 minutes each followed by washing in 1xTBS-T 4 times for 5 minutes each. Stripped blots were blocked overnight at 4 °C in 5 %(w/v) milk in TBS-T before probing with anti-BAK1 antibodies (Supplementary Table S4).

Immune assays

For MAPK assays, seedlings were grown in 24-well plates (one seedling per well) as described above. Growth media was removed by inverting plates on paper towels and individual seedlings were treated as indicated in the figure legends. Two seedlings were pooled for each treatment/time point. Total proteins were extracted as described above, normalized by Bradford assay and analyzed by SDS-PAGE and immunoblotting with anti-p44/42 antibodies (Supplementary Table S3).

For analysis of the PAMP-induced oxidative burst, leaf discs from 3- to 4-week-old plants were collected into white 96-well plates using a 4 mm biopsy punch and were allowed to rest in sterile ultrapure water overnight. The next day, the water was removed and replaced with a solution containing 100 nM elf18, 1 mM luminol, and 10 µg/mL HRP (in sterile ultrapure water). Luminescence was collected for 70 minutes using a Photek system equipped with a photon counting camera.

For seedling growth inhibition assays, 4-day-old seedlings were transferred to 48-well plates (one seedling per well) containing MS with mock (sterile ddH_20) or elf18 at the concentration indicated in figure captions. Seedlings were grown for 10 days in the treatment solution and the weights of individual seedlings were recorded using an analytical balance.

PR1 accumulation was evaluated by immunoblotting protein extracts from leaves treated with elf18. Three leaves from 3- to 4-week-old plants were pressure infiltrated with either mock (sterile ultrapure water) or 1 µM elf18. After 24 hours, leaves were removed by cutting with sharp scissors and were snap-frozen in liquid nitrogen in 1.5-mL tubes and were then pulverized with a nitrogen-cooled plastic micropestle. Total proteins were extracted by grinding at 2,000 rpm in extraction buffer (see above) using a micropestle fixed to a rotary mixer. Protein extracts were normalized by Bradford assay and were

analyzed by SDS-PAGE and immunoblotting using anti-PR1 antibodies (Supplementary Table S4).

Agrobacterium-mediated transient transformation and induced resistance assays

Analysis of GUS activity following transient transformation of Arabidopsis leaves was performed as previously described (1, 3). Briefly, *Agrobacterium tumefaciens* GV3101 carrying the pBIN19g:GUS (containing a potato intron) plasmid was infiltrated into the leaves of 3- to 4-week-old plants at an OD_{600} of 0.4. After 5 days, infiltrated leaves were removed by cutting with sharp scissors and were snap-frozen in liquid nitrogen in 1.5-mL tubes. Total proteins were extracted in GUS assay buffer (3) and GUS activity was measured after 30 minutes of incubation in the presence of 1 mM 4-methylumbelliferyl-β-D-glucuronide (MUG, Sigma Aldrich). Reactions were stopped by the addition of four volumes of 0.2 M Na_2CO_3 and fluorescence was measured in a Biotek Synergy microplate reader with excitation and emission wavelengths of 365 nm and 455 nm, respectively. The amount of 4-methylumbelliferone (4-MU) produced was measured against a standard curve of 4-MU prepared in methanol.

Induced resistance assays were performed as described previously (4). Briefly, 3 leaves each of 5-week-old plants grown on soil were infiltrated with a solution of 1 µM elf18 or mock (sterile ddH_20) in the morning. The following morning treated leaves were re-infiltrated with a suspension of approximately 10^8 *Pseudomonas syringae* pv. tomato DC3000 (*Pto* DC3000) per mL (OD_{600}=0.0002). Plants were left uncovered for two days, after which two leaf discs were harvested per treated leaf and six leaf discs pooled per plant. Colony forming units (CFU) per cm^2 were counted through serial dilution, and statistics performed on $log_{10}(CFU/cm^2)$ in R (5). ANOVA revealed significant effects of genotype and treatment, as well as a significant interaction (p<2.2x10-16). The effect of

treatment within each genotype was estimated through estimated marginal means (package emmeans) with no correction for multiple testing (6).

Co-immunoprecipitation and IP kinase assays

Protein extracts containing GFP-tagged EFR or site-directed mutants were incubated with 20 µL of GFP-Trap beads (Chromotek) or GFP-clamp beads (7) as indicated in figure captions for 2 hours with gentle mixing at 4 °C to immuno-precipitate receptor complexes. The beads were sedimented by centrifugation at 1000 x *g* for 5 minutes at 4 °C and were subsequently suspended in 1 mL of extraction buffer (see above). The beads were sedimented at 1,000 x *g* for 1 minute and suspended in 1 mL of extraction buffer three more times for a total of four washes. After the last wash was removed, beads were suspended in 2X Laemmli SDS-PAGE loading buffer followed by heating at 80 °C for 10 minutes. For IP-kinase assays, beads were equilibrated in 1 mL kinase assay buffer containing 50 mM HEPES-NaOH pH 7.2, 100 mM NaCl, 5 mM each $MgCl_2$ and $MnCl_2$, and 5 %(v/v) glycerol. The total volume of kinase assay buffer and beads was split in two and half was immediately prepared for SDS-PAGE by removal of the kinase assay buffer and heating of the beads at 80 °C for 10 minutes in 2X Laemmli SDS-PAGE loading buffer. The second half was used for an *in vitro* on-bead kinase assay.

After removal of the equilibration buffer volume, the beads were suspended in 20 µL of fresh kinase assay buffer containing 1 µM ATP and 5 µCi of γ^{32}P-ATP. Kinase reactions were incubated at 30 °C for 30 minutes with shaking at 800 rpm in an Eppendorf Thermomixer. The reactions were stopped by the addition of 10 µL of 3X Laemmli SDS-PAGE loading buffer and heating at 80 °C for 10 minutes. Twenty-five microliters of each reaction were loaded into a 10 %(v/v) SDS-PAGE gel and proteins were separated for 90-

100 minutes at 120 V followed by transfer to PVDF. A storage-phosphor screen was exposed overnight with the PVDF membrane and exposed screens were visualized using a Typhoon imager (GE Lifesciences).

Mass spectrometric analysis

Samples were prepared and analysed by LC-MS/MS as previously described (8, 9). The mass spectrometry proteomics data have been deposited to the ProteomeXchange Consortium via the PRIDE (10) partner repository with the dataset identifier PXD025597 and 10.6019/PXD025597.

Homology modelling and visualization

The homology model of the EFR protein kinase domain (residues 712-1001) was generated using MODELLER (11) implemented in Chimera (v1.15; (12). The published BAK1 protein kinase domain structures 3UIM (13) and 3TL8 (14) were used as templates for the model.

Software

Figures were prepared using Inkscape (v0.92.3) and GIMP (v2.10.4). Raw immunoblots were converted to TIFF format using BioRad Image Lab (v6.0.1). Plotting and statistical analysis was carried out in GraphPad Prism (v8.3.0 and 9.0.0). Multiple sequence alignments were generated using the ClustalO algorithm in Jalview (v2.10.5). The version of R and emmeans used for analysis of induced resistance data were 4.0.2 and 1.5.3, respectively.

References

1. Zipfel C, Kunze G, Chinchilla D, Caniard A, Jones JDG, Boller T, et al. Perception of the bacterial PAMP EF-Tu by the receptor EFR restricts *Agrobacterium*-mediated transformation. Cell. 2006 May 19;125(4):749–60.
2. Clough SJ, Bent AF. Floral dip: a simplified method for *Agrobacterium*-mediated transformation of *Arabidopsis thaliana*. The Plant Journal. 1998 Dec 1;16(6):735–43.
3. Jefferson RA, Kavanagh TA, Bevan MW. GUS fusions: beta-glucuronidase as a sensitive and versatile gene fusion marker in higher plants. EMBO J. 1987 Dec 20;6(13):3901–7.
4. Zipfel C, Robatzek S, Navarro L, Oakeley EJ, Jones JDG, Felix G, et al. Bacterial disease resistance in Arabidopsis through flagellin perception. Nature. 2004 Apr 15;428(6984):764–7.
5. R Foundation for Statistical Computing RCT. R: A Language and Environment for Statistical Computing . Vienna, Austria: R Foundation for Statistical Computing; 2020.
6. Lenth RV. emmeans:Estimated Marginal Means, aka Least-Squares Means . 2020.
7. Hansen S, Stüber JC, Ernst P, Koch A, Bojar D, Batyuk A, et al. Design and applications of a clamp for Green Fluorescent Protein with picomolar affinity. Sci Rep. 2017 Nov 24;7(1):16292.
8. Ntoukakis V, Mucyn TS, Gimenez-Ibanez S, Chapman HC, Gutierrez JR, Balmuth AL, et al. Host inhibition of a bacterial virulence effector triggers immunity to infection. Science. 2009 May 8;324(5928):784–7.
9. Piquerez SJM, Balmuth AL, Sklenář J, Jones AME, Rathjen JP, Ntoukakis V. Identification of post-translational modifications of plant protein complexes. J Vis Exp. 2014 Feb 22;(84):e51095.
10. Perez-Riverol Y, Csordas A, Bai J, Bernal-Llinares M, Hewapathirana S, Kundu DJ, et al. The PRIDE database and related tools and resources in 2019: improving support for quantification data. Nucleic Acids Res. 2019 Jan 8;47(D1):D442–D450.
11. Eswar N, Webb B, Marti-Renom MA, Madhusudhan MS, Eramian D, Shen M-Y, et al. Comparative protein structure modeling using MODELLER. Curr Protoc Protein Sci. 2007 Nov;Chapter 2:Unit 2.9.
12. Pettersen EF, Goddard TD, Huang CC, Couch GS, Greenblatt DM, Meng EC, et al. UCSF Chimera—a visualization system for exploratory research and analysis. J Comput Chem. 2004 Oct;25(13):1605–12.
13. Yan L, Ma Y, Liu D, Wei X, Sun Y, Chen X, et al. Structural basis for the impact of phosphorylation on the activation of plant receptor-like kinase BAK1. Cell Res. 2012 Aug;22(8):1304–8.
14. Cheng W, Munkvold KR, Gao H, Mathieu J, Schwizer S, Wang S, et al. Structural analysis of Pseudomonas syringae AvrPtoB bound to host BAK1 reveals two similar kinase-interacting domains in a type III Effector. Cell Host Microbe. 2011 Dec 15;10(6):616–26.

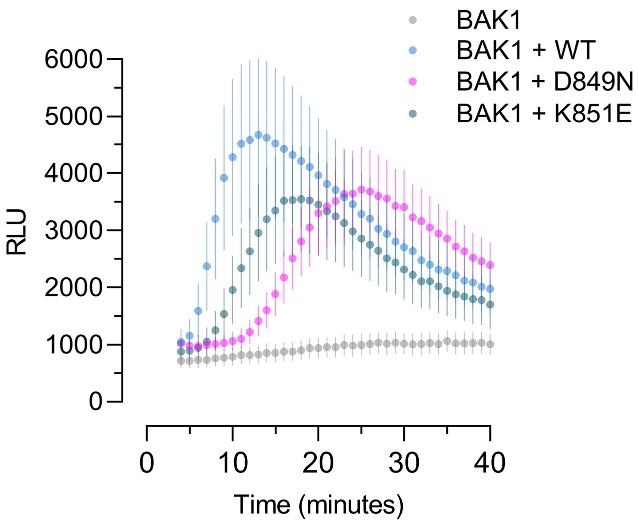

Figure S1. Analysis of the elf18-induced oxidative burst in *N. benthamiana* leaves after transient expression of EFR-GFP or catalytic site mutants. Each EFR variant was co-expressed with Arabidopsis BAK1, and expression of BAK1 alone served as a control for EFR-dependence of the elf8-triggered oxidative burst. Leaf discs were treated with 100 nM elf18 and luminescence was measured for 35 minutes. Points are mean with standard error from six replicate infiltrations.

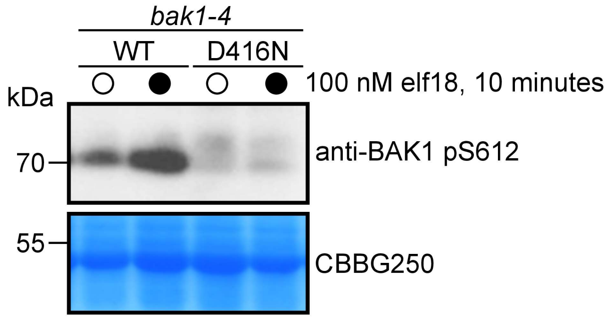

Figure S2. elf18-induced BAK1 S612 phosphorylation depends on BAK1 protein kinase activity. Immunoblot analysis of elf18-induced phosphorylation of BAK1 (anti-BAK1-pS612) in 12-day-old transgenic seedlings expressing either wild-type (WT) BAK1 or the kinase-dead D416N mutant. Seedlings were treated with mock (open circles) or 100 nM elf18 (closed circles) for 10 minutes. Coomassie stain is shown as loading control (CBBG250). The experiment was performed twice times with similar results.

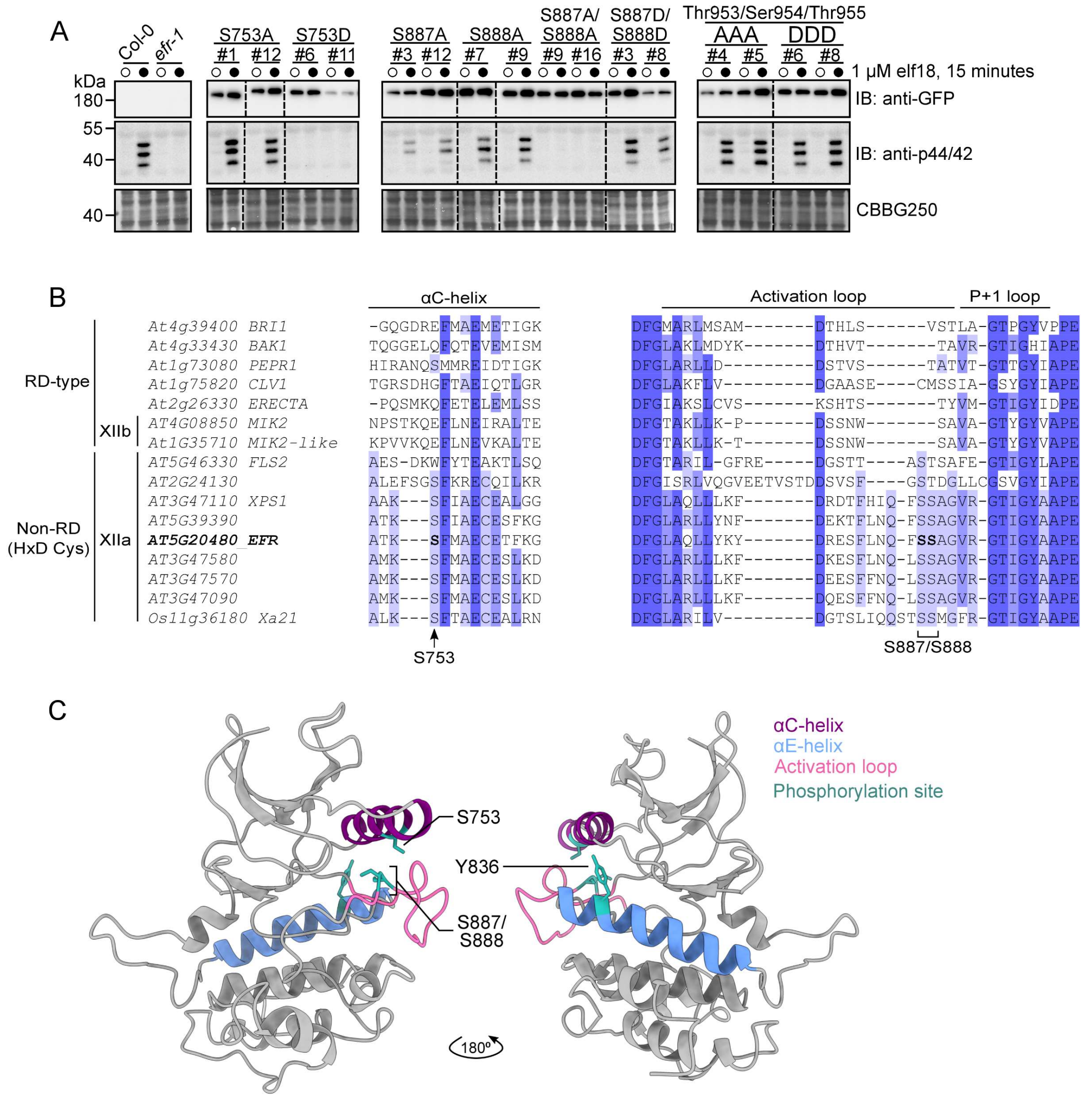

Figure S3. Screen of phosphorylation site mutants for MAPK activation and conservation of regulatory phosphorylation sites. A, Immunoblot analysis of MAPK phosphorylation (anti-p44/42) after treatment with mock (open circles) or 1 µM elf18 (closed circles) for 15 minutes in 12-day-old seedlings for non-phosphorylatable (Ala) and phospho-mimic (Asp) mutants of selected EFR phosphorylation sites. Anti-GFP immunoblotting indicates accumulation of the receptor in transgenic plants. Coomassie stained immunoblots are shown as a loading control (CBBG250). **B**, Multiple sequence alignment of Arabidopsis LRR-RKs from subfamily XII with other well-known RKs. Regions of the alignment representing the αC-helix and the activation loop were extracted from an alignment of cytoplasmic domains to reveal conservation of novel regulatory EFR phosphorylation sites. **C**, Homology model of the EFR protein kinase domain showing the location of regulatory phosphorylation sites within important subdomains of the protein kinase.

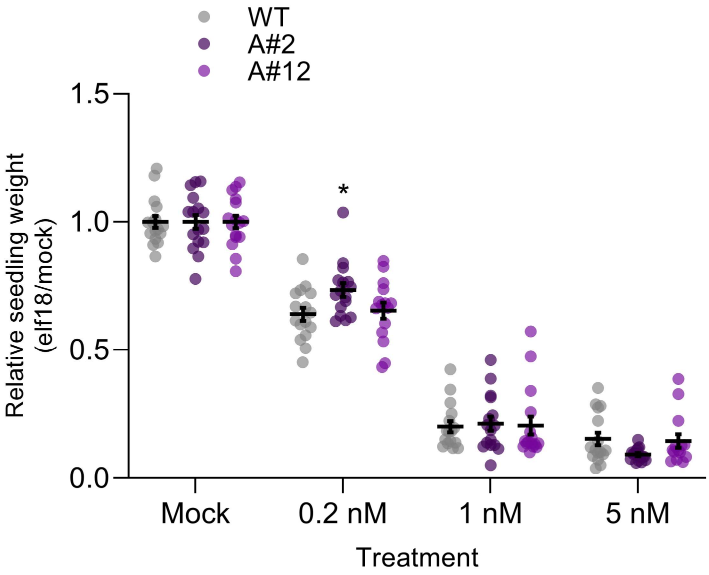

Figure S4. EFR^{S753A} is not hyper-sensitive to elf18. Seedling growth of the indicated genotypes in the presence of different concentrations of elf18. Data are shown relative to mock treated seedlings for each genotype. Individual data points with mean and standard error are shown. Asterisk indicates statistical difference from WT under a given treatment (Two-way ANOVA, $p<0.000001$, $n=16$, Dunnett's multiple comparison test). The experiment was repeated three times with similar results. Results from a representative experiment are shown.

Supplementary Table S1. Representative phosphopeptides identified on immunopurified EFR-GFP**.**

Phosphosite	Treatment[a]	Mascot ion score	Peptide[b,c]	Observed mass	Actual mass	Charge	Δ ppm
Ser683‡	m/e	62.46	K.NNA**pS**DGNPSDSTTLGmFHEK.V	1109.44	2216.87	2	0.8529
Ser688†‡	e	46.68	K.NNASDGNP**pS**DSTTLGmFHEK.V	739.96	2216.86	3	-2.559
Ser690†‡§	m/e	54.29	K.KNNASDGNPSD**pS**TTLGmFHEK.V	782.66	2344.96	3	0.2752
Thr691†	m	58.22	K.NNASDGNPSDS**pT**TLGmFHEK.V	1109.44	2216.87	2	1.078
Ser707§	e	55.29	K.VSYEELH**pS**ATSR.F	729.82	1457.62	2	0.6215
Thr709	e	75.46	K.VSYEELHSA**pT**SR.F	729.82	1457.62	2	-0.6399
Ser753‡	e	25.75	K.HGATK**pS**FmAEcETFK.G	613.92	1838.74	3	1.316
Ser781§	e	31.6	K.LITVCSSLD**pS**EGNDFR.A	946.91	1891.81	2	-0.52
Ser888‡§	e	54.47	K.YDRESFLNQFS**pS**AGVR.G	978.44	1954.86	2	1.68
Thr953	e	65.51	K.SILSGc**pT**SSGGSNAIDEGLR.L	1030.95	2059.89	2	2.563
Ser954‡	m/e	84.22	K.SILSGcT**pS**SGGSNAIDEGLR.L	1030.95	2059.89	2	-1.348
Ser1010†	m/e	47.6	K.TTITE**pS**PR.D	492.72	983.43	2	1.348

a, in this study; m, mock treatment; e, 100 nM elf18

b, boldface indicates the phosphorylated Ser or Thr; lowercase 'm' or 'c' indicates oxidized Met and carbamidomethyl Cys, respectively

c, all phosphopeptide spectra were manually inspected to verify site localizations

†, identified in Mergner *et al*. 2020 Arabidopsis phosphoproteome

‡, *in vitro* autophosphorylation site according to Wang *et al*. 2014

§, *in vitro* transphosphorylation by BAK1 according to Wang *et al*. 2014

Supplementary Table S2. Reported requirements for catalytic activity of plant receptor kinases.

Protein name	Species	Sequence identifier[a]	Ectodomain	ePK type	Background	Mutation	Expression[b]	Details	PubmedID
EFR	*A. thaliana*	AT5G20480	LRR	non-RD	-	D849N	n.d.	Kinase activity required for elf18-induced oxidative burst in *N. benthamiana* (transient expression under 35S promoter)	21593986
FLS2	*A. thaliana*	AT5G46330	LRR	non-RD	Col-0	G1064R	n.d.	*fls2-17*; mutant also has reduces flg22 binding, suggesting reduced accumulation of FLS2(G1064R)	11340188
FLS2	*A. thaliana*	AT5G46330	LRR	non-RD	*fls2-24*	K898M	protein	Kinase activity required for activation of MAPK cascades in protoplasts	11875555
FLS2	*A. thaliana*	AT5G46330	LRR	non-RD	*fls2 bak1-4*	D868A/K898A	n.d.	Kinase activity required for function of a chimeric receptor in protoplasts	24130196
FLS2	*A. thaliana*	AT5G46330	LRR	non-RD	*fls2-101*	D997A	n.d.	Kinase activity required for flg22-induced seedling growth inhibition	22388452
Xa21	*O. sativa*	Os11g0569733	LRR	non-RD	TP309	K736E	protein*	Kinase-dead mutant confers partial resistance to Xoo PXO99A	20616165
ANJ	*A. thaliana*	AT5G59700	Malectin	RD	*herk1 anj*	D609N/K611R	-	Kinase-dead mutant complements pollen tube overgrowth phenotype	31867824
BAK1	*A. thaliana*	AT4G33430	LRR	RD	*bak1-4*	K317E/G537R	protein	Kinase activity required for flg22-induced oxidative burst	20103591
BAK1	*A. thaliana*	AT4G33430	LRR	RD	*bak1-4*	D416N	protein	Kinase-dead mutant dominant negative for elf18-induced oxidative burst	21593986
BAK1	*A. thaliana*	AT4G33430	LRR	RD	*bak1*	K317E	-	Kinase activity required for ABA-induced stomatal closure	26724418
BAK1	*A. thaliana*	AT4G33430	LRR	RD	Col-0	D434N	protein	No dominant negative effect on BR signaling; long *BAK1* splice variant and native promoter	21464298
BAK1	*A. thaliana*	AT4G33430	LRR	RD	*bak1*	K317M	protein	Loss of flg22-dependent CERK1 phosphorylation, but not MAPK phosphorylation (protoplasts)	31830443
BAK1	*A. thaliana*	AT4G33430	LRR	RD	*bri1-5*	K317E	n.d	Kinase-dead mutant confers dominant negative BR phenotype (over-expression)	22253607
BAK1	*A. thaliana*	AT4G33430	LRR	RD	*bri1-301*	K317E	protein	Kinase-dead mutant confers dominant negative BR phenotype (over-expression)	28461403
BAK1	*A. thaliana*	AT4G33430	LRR	RD	*bri1-702*	K317E	protein	Kinase-dead mutant confers dominant negative BR phenotype (over-expression)	28461403
BAK1	*A. thaliana*	AT4G33430	LRR	RD	*bak1-5*	K317E	protein	Kinase activity required for response to extracellular $NADP^+$	31641112
BIR1	*A. thaliana*	AT5G48380	LRR	RD	*bir1-1*	K331E	n.d.	Kinase-dead mutant does not complement loss of BIR1 function	19616764
BKK1	*A. thaliana*	AT2G13790	LRR	RD	*bri1-5*	K322E	n.d.	Kinase-dead mutant confers dominant negative BR phenotype (over-expression)	22253607
BRI1	*A. thaliana*	AT4G39400	LRR	RD	*bri1-5*	K911E	protein	Kinase-dead mutant fails to complement *bri1-5* dwarf phenotype	15894717
BRI1	*A. thaliana*	AT4G39400	LRR	RD	*bri1-5*	K911E	protein	Kinase activity required for *in vivo* BL-dependent phosphorylation of BAK1	18694562
BRI1	*A. thaliana*	AT4G39400	LRR	RD	Col-0	P1050S	-	*bri1-702*; weak BR phenotype similar to *bri1-5* and partial loss of *in vitro* autophosphorylation	28461403
BRI1	*A. thaliana*	AT4G39400	LRR	RD	Col-0	G989I	-	*bri1-301*; weak BR phenotype	28461403
BRI1	*A. thaliana*	AT4G39400	LRR	RD	Col-0	R983G	-	*bri1-708*; *BRI1* null phenotype	28461403
BRI1	*A. thaliana*	AT4G39400	LRR	RD	Col-0	E1056K	-	*bri1-703*; *BRI1* null phenotype	28461403
BRI1	*A. thaliana*	AT4G39400	LRR	RD	Col-0	D1027N	-	*bri1-704*; *BRI1* null phenotype	28461403
BRI1	*A. thaliana*	AT4G39400	LRR	RD	Col-0	R983N	-	*bri1-8*	28461403
BRI1	*A. thaliana*	AT4G39400	LRR	RD	Col-0	R983Q	-	*bri1-108*	10938344
BRI1	*A. thaliana*	AT4G39400	LRR	RD	Col-0	A909T	-	*bri1-1*; possible ATP-binding mutant, severe BR insensitive phenotype	8754677
CERK1	*A. thaliana*	AT3G21630	LysM	RD	*cerk1-2*	K350N	protein	Kinase activity required for chitin-induced oxidative burst	20610395
CERK1	*A. thaliana*	AT3G21630	LysM	RD	*cerk1*	ΔRD, K350N	protein	Kinase activity required for Y428 phosphorylation	29396039
CERK1	*O. sativa*	Os08g0538300	LysM	RD	*oscerk1*	D418V	protein	Kinase activity required for LPS-induced oxidative burst	29194653
CERK1	*O. sativa*	Os08g0538300	LysM	RD	*oscerk1*	D418V	protein	Kinase activity required for chitin elicitor response in suspension culture	24964058
CRK28	*A. thaliana*	AT4G21400	DUF26	RD	Col-0	K377N	protein	Kinase activity required for reduced stature in Arabidopsis and cell death induced by over-expression in *N. benthamiana*	27852951
CRK36	*A. thaliana*	AT4G04490	DUF26	RD	Col-0	K368E		Over-expression of a kinase-dead mutant in Col-0 supresses flg22-induced stomatal closure	29163585
FER	*A. thaliana*	AT3G51550	Malectin	RD	*fer-4*	K595R	transcript	Kinase-dead mutant partialls complements seedling growth and RALF1 response, and fully complements of ovule fertilization	29904923
FER	*A. thaliana*	AT3G51550	Malectin	RD	*fer-4*	K595R	protein*	Kinase-dead mutant partially complements mechanical responses (surface pH, root tip angle)	25127214
FER	*A. thaliana*	AT3G51550	Malectin	RD	*fer-1*	K595R,A,E	protein*	Kinase-dead mutants complement ovule fertilization phenotype	25490905
FER	*A. thaliana*	AT3G51550	Malectin	RD	*fer-4*	K595R	protein	Kinase activity not required for scaffolding of FLS2-BAK1 complex (bioRxiv; 10.1101/2020.07.20.212233)	-
HAESA	*A. thaliana*	AT4G28490	LRR	RD	*hae-3 hsl2-3*	K711E	proteinb	Kinase activity required for floral organ abscission	26784444
HERK	*A. thaliana*	AT3G46290	Malectin	RD	*herk1 anj*	D606N/K608R	-	Kinase-dead complement pollen tube overgrowth phenotype	31867824
LecRK	*H. bulbosum*	QDJ58010	L-Lectin	RD	-	D512A	n.d.	Kinase-dead mutant not trigger oxidative burst or cell death in *N. benthamiana*	31712760
LecRK-VI.2	*A. thaliana*	AT5G01540	L-Lectin	RD	*lecrk-vi.2-2*	D494N	protein	Kinase activity required for response to extracellular $NADP^+$	31641112
LecRK-IX.1	*A. thaliana*	At5g10530	L-Lectin	RD	Col-0	D459N, R458A/D459A	transcript	Kinase-dead does not induce cell death	26011556
LecRK-IX.2	*A. thaliana*	At5g65600	L-Lectin	RD	Col-0	D475N, R474A/D475A	transcript	Kinase-dead does not induce cell death	26011556
LecRK-IX.2	*A. thaliana*	At5g65600	L-Lectin	RD	-	K379R, K477E, D532N	protein	Kinase activity required to trigger cell death in *N. benthamiana*	28696275
LecRK-S.7	*O. sativa*	Os02g0459600	L-Lectin	RD	cr-oslecrk-S.7	K418E, E560K	n.d.	Kinase activity required for fertility	31833176
LecRK-S.7	*O. sativa*	Os02g0459600	L-lectin	RD	*oslecrk5*	K418E	n.d.	Kinase activity required for callose deposition during microsporogenesis	32270203
LORE	*A. thaliana*	AT1G61380	G-Lectin	RD	*lore*	K516E, D613V	protein	Kinase activity required to trigger immune responses to 3-OH-C10:0	31922267
LRK1	*N. benthamiana*	B3XWM9	L-Lectin	RD	-	K314R	protein	Kinase activity required for INF1-induced phosphorylation	18682978
LYK3	*M. truncatula*	Q6UD73	LysM	RD	-	K349A	n.d.	Kinase activity required for cell death triggered in *N. benthamiana* after co-infiltration with MtNFP	23750228
MIK2	*A. thaliana*	AT4G08850	LRR	RD	-	K802A	n.d.	Kinase-dead mutant does not respond to EnFOE following transient expression in *N. benthamiana*	33253435
NFR1	*L. japonicus*	Q70KR8	LysM	RD	*nfr1-3*	K350E	n.d.	Kinase-dead mutant does not complement loss of nodulation in *nfr1-3*	21265894
NFR5	*L. japonicus*	Q70KR1	LysM	RD	*nfr5-2*	K339E	n.d.	Kinase dead mutant complements loss of nodulation in *nfr5-2*	21265894
NIK1	*A. thaliana*	AT5G16000	LRR	RD	*nik1-1*	R340A	protein	Kinase activity not required for suppression of FLS2-BAK1 complex formation; unconventional mutation	31676803
P2K2	*A. thaliana*	AT3G45430	L-Lectin	RD	*pk21-3*	D467N,D525N	n.d.	Kinase activity required for response to extracellular ATP (Ca^{2+} influx)	32345768
PRK2	*A. thaliana*	AT2G07040	LRR	RD	Col-0	K366R	protein*	Kinase-dead mutant disrupts pollen tube integrity similar to wild-type protein	24136420
PSKR1	*A. thaliana*	AT2G02220	LRR	RD	*r1r2*	K762E	transcript	Kinase dead mutant does not complement reduced root elongation phenotype of *r1r2*	24495073
RPK1	*A. thaliana*	AT1G69270	LRR	RD	*rpk1*	K289E	n.d.	Kinase-dead mutant is insensitive to ABA for stomatal closure	31665747
SERK1	*A. thaliana*	AT1G71830	LRR	RD	*bri1-5*	K330E	n.d	Kinase-dead mutant confers dominant negative BR phenotype (over-expression)	22253607
SERK2	*A. thaliana*	AT1G34210	LRR	RD	*bri1-5*	K333E	n.d	Kinase-dead mutant confers dominant negative BR phenotype (over-expression)	22253607
SERK5	*A. thaliana*	AT2G13800	LRR	RD	*bri1-5*	K301E	n.d.	Kinase-dead mutant confers dominant negative BR phenotype (over-expression); Ler allele	26528315
SIF2	*A. thaliana*	AT1G51850	LRR-Mal	RD	*sif2-1*	D683N	transcript	Kinase activity required for complementation of anti-bacterial immunity in *sif2-1*	32327536
SIT1	*O. sativa*	Os02g0640500	L-Lectin	RD	Col-0	K386E, D842A	protein	Kinase activity required for over-expression induced salt sensitivity in Arabidopsis	24907341
SOBIR1	*A. thaliana*	AT2G31880	LRR	RD	-	D489N	protein	Kinase activity required to trigger cell death in *N. benthamiana*	30407725
SOBIR1	*A. thaliana*	AT2G31880	LRR	RD	-	D489N	-	Kinase activity required to trigger cell death in *N. benthamiana*	28876174
SOBIR1	*A. thaliana*	AT2G31880	LRR	RD	Col-0	E407K	-	*evr-2*; EMS mutant allele characterized by loss of kinase activity	20081191
SRKb	*A. lyrata*	Q9AVE0	S-locus	RD	*rdr6-11*	K529R	transcript	Kinase activity require for self-incompatibility response.	19767457
TMS10; SERK4	*O. sativa*	Os02g0283800	LRR	RD	*tms10*	K312E	protein	Kinase-dead mutant fails to rescue male fertility defect	29087306

a, Araport 11 (*A. thaliana*), Rice Annotation Project Database (*O. sativa*), or Uniprot (all others)

b, n.d., no data; *, assayed by fluorescence microscpoy

Supplementary Table S3. Oligonucleotide primers used in this study

Primer name	Sequence (5' - 3')	Purpose
LBb1.3	ATTTTGCCGATTTCGGAAC	*efr-1* T-DNA genotyping
SALK_044334-LP	TGGAAATAACTCGTCCAGTGG	*efr-1* T-DNA genotyping
EFRintronR3	GAAACAACACACAATGAGGTTGG	*efr-1* T-DNA genotyping
EFR-CD_BamHI-F	ATCGGGATCCAACAATGCCAGTGATGGTAACCC	MBP fusion cloning
EFR-CD_SalI-R	ATCGGTCGACCTACATAGTATGCATGTCCG	MBP fusion cloning
MBPseq_f	TCGTCAGACTGTCGATGAAGCCCTG	pMAL-c4E sequcning primer
M13_forward	GTAAAACGACGGCCAGT	pMAL-c4E sequcning primer
EFRpro-F	CCAAGCTGAGCTGAATTCATCTAGACGATTAAGTAATTGAGC	Complementation construct cloning; InFusion
EFRpro-R	GTGAAAAGGACAGCTTCATGTCGATTATAAAAAGATAAAAG	Complementation construct cloning; InFusion
EFRcds-F	CTTTTATCTTTTTATAATCGACATGAAGCTGTCCTTTTCAC	Complementation construct cloning; InFusion
EFRcds-R	CCTTGCTCACCATGGATCCCATAGTATGCATGTCCG	Complementation construct cloning; InFusion
epiGreen-F	TGACGCACAATCCCACTATC	Complementation constuct sequencing
EFRpro-Seq1	ATCTAGACGATTAAGTAATTGAGC	Complementation constuct sequencing
EFRpro-Seq2	CGCATTTAATTGCCCCACG	Complementation constuct sequencing
EFRpro-Seq3	GTCGATTATAAAAAGATAAAAGAAAGGTTC	Complementation constuct sequencing
EFR-Seq1	CTTGCCATTCTGGATCTTAGC	Complementation constuct sequencing
EFR-Seq2	ACCATTCCTCATGACATCG	Complementation constuct sequencing
EFR-Seq3	AGTTTGAGGGAAGGGTGC	Complementation constuct sequencing
EFR-Seq4	AAGGGTAAACGATCACTCG	Complementation constuct sequencing
eGFP-SeqR	CAGATGAACTTCAGGGTCAGC	Complementation constuct sequencing
EFR_D849N_s	CATGACCCTGTAGCTCACTGTAATATTAAGCCAAGCAACATTC	Site-directed mutagenesis
EFR_D849N_as	GAATGTTGCTTGGCTTAATATTACAGTGAGCTACAGGGTCATG	Site-directed mutagenesis
EFR_K851E_s	GTAGCTCACTGTGATATTGAGCCAAGCAACATTCTTCTAG	Site-directed mutagenesis
EFR_K851E_as	CTAGAAGAATGTTGCTTGGCTCAATATCACAGTGAGCTAC	Site-directed mutagenesis
EFR_S753A_s	CCTAAAGCATGGAGCGACGAAAGCCTTTATGGCGGAATGTGAAACC	Site-directed mutagenesis
EFR_S753A_as	GGTTTCACATTCCGCCATAAAGGCTTTCGTCGCTCCATGCTTTAGG	Site-directed mutagenesis
EFR_S753D_s	CCTAAAGCATGGAGCGACGAAAGCCTTTATGGCGGAATGTGAAACC	Site-directed mutagenesis
EFR_S753D_as	GGTTTCACATTCCGCCATAAAGGCTTTCGTCGCTCCATGCTTTAGG	Site-directed mutagenesis
EFR_S887A_s	CGAGAATCCTTTCTAAACCAGTTTGCTTCTGCTGGTGTCAGAGGCACC	Site-directed mutagenesis
EFR_S887A_as	GGTGCCTCTGACACCAGCAGAAGCAAACTGGTTTAGAAAGGATTCTCG	Site-directed mutagenesis
EFR_S888A_s	CGAGAATCCTTTCTAAACCAGTTTAGTACTGCTGGTGTCAGAGGCACC	Site-directed mutagenesis
EFR_S888A_as	GGTGCCTCTGACACCAGCAGTACTAAACTGGTTTAGAAAGGATTCTCG	Site-directed mutagenesis
EFR_S887A/S888A_s	CGAGAATCCTTTCTAAACCAGTTTGCTACTGCTGGTGTCAGAGGCACC	Site-directed mutagenesis
EFR_S887A/S888A_as	GGTGCCTCTGACACCAGCAGTAGCAAACTGGTTTAGAAAGGATTCTCG	Site-directed mutagenesis
EFR_S887D/S888D_s	CGAGAATCCTTTCTAAACCAGTTTGATGATGCTGGTGTCAGAGGCACC	Site-directed mutagenesis
EFR_S887D/S888D_as	GGTGCCTCTGACACCAGCATCATCAAACTGGTTTAGAAAGGATTCTCG	Site-directed mutagenesis
EFR_TST-AAA_s1	GTCTATATTATCGGGTTGCGCGAGCAGTGGAGGCAGCAACGCCATTG	Site-directed mutagenesis
EFR_TST-AAA_as1	CAATGGCGTTGCTGCCTCCACTGCTCGCGCAACCCGATAATATAGAC	Site-directed mutagenesis
EFR_TST-AAA_s2	GTCTATATTATCGGGTTGCGCGGCCAGTGGAGGCAGCAACGCCATTG	Site-directed mutagenesis
EFR_TST-AAA_as2	CAATGGCGTTGCTGCCTCCACTGGCCGCGCAACCCGATAATATAGAC	Site-directed mutagenesis
EFR_TST-AAA_s3	GTCTATATTATCGGGTTGCGCGGCCGCTGGAGGCAGCAACGCCATTG	Site-directed mutagenesis
EFR_TST-AAA_as3	CAATGGCGTTGCTGCCTCCAGCGGCCGCGCAACCCGATAATATAGAC	Site-directed mutagenesis
EFR_TST-DDD_s1	GTCTATATTATCGGGTTGCGACAGCAGTGGAGGCAGCAACGCCATTG	Site-directed mutagenesis
EFR_TST-DDD_as1	CAATGGCGTTGCTGCCTCCACTGCTGTCGCAACCCGATAATATAGAC	Site-directed mutagenesis
EFR_TST-DDD_s2	GTCTATATTATCGGGTTGCGACGACAGTGGAGGCAGCAACGCCATTG	Site-directed mutagenesis
EFR_TST-DDD_as2	CAATGGCGTTGCTGCCTCCACTGTCGTCGCAACCCGATAATATAGAC	Site-directed mutagenesis
EFR_TST-DDD_s3	GTCTATATTATCGGGTTGCGACGACGATGGAGGCAGCAACGCCATTG	Site-directed mutagenesis
EFR_TST-DDD_as3	CAATGGCGTTGCTGCCTCCATCGTCGTCGCAACCCGATAATATAGAC	Site-directed mutagenesis

Supplementary Table S4. Antibodies used in this study.

Antibody	Source	Catalog #	Dilution	Incubation time	Reference
anti-GFP-HRP (clone B-2)	Santa Cruy Biotechnology	sc9996 HRP	1:2000	12-16 hours	n/a
anti-p44/42	Cell Signaling Technology	9101L	1:2000	2 hours	n/a
anti-BAK1	Custom	n/a	1:10000	12-16 hours	Perraki *et al.* [a]
anti-BAK1 pS612	Custom	n/a	1:4000	2 hours	Perraki *et al.* [a]
anti-PR1	Agrisera	AS10 687	1:2500	2 hours	n/a

a, Perraki *et al* . (2018) Nature **561** 248-252
